# Fifty Generations of Amitosis: Tracing Asymmetric Allele Segregation in Polyploid Cells with Single-Cell DNA Sequencing

**DOI:** 10.3390/microorganisms9091979

**Published:** 2021-09-17

**Authors:** Valerio Vitali, Rebecca Rothering, Francesco Catania

**Affiliations:** Institute for Evolution and Biodiversity, University of Münster, 48149 Münster, Germany; rebecca.rothering@uni-muenster.de (R.R.); francesco.catania@uni-muenster.de (F.C.)

**Keywords:** amitosis, single-cell DNA sequencing, developmental plasticity, somatic mutations, polyploidy

## Abstract

Amitosis is a widespread form of unbalanced nuclear division whose biomedical and evolutionary significance remain unclear. Traditionally, insights into the genetics of amitosis have been gleaned by assessing the rate of phenotypic assortment. Though powerful, this experimental approach relies on the availability of phenotypic markers. Leveraging *Paramecium tetraurelia*, a unicellular eukaryote with nuclear dualism and a highly polyploid somatic nucleus, we probe the limits of single-cell whole-genome sequencing to study the consequences of amitosis. To this end, we first evaluate the suitability of single-cell sequencing to study the AT-rich genome of *P.* *tetraurelia*, focusing on common sources of genome representation bias. We then asked: can alternative rearrangements of a given locus eventually assort after a number of amitotic divisions? To address this question, we track somatic assortment of developmentally acquired Internal Eliminated Sequences (IESs) up to 50 amitotic divisions post self-fertilization. To further strengthen our observations, we contrast empirical estimates of IES retention levels with in silico predictions obtained through mathematical modeling. In agreement with theoretical expectations, our empirical findings are consistent with a mild increase in variation of IES retention levels across successive amitotic divisions of the macronucleus. The modest levels of somatic assortment in *P.* *tetraurelia* suggest that IESs retention levels are largely sculpted at the time of macronuclear development, and remain fairly stable during vegetative growth. In forgoing the requirement for phenotypic assortment, our approach can be applied to a wide variety of amitotic species and could facilitate the identification of environmental and genetic factors affecting amitosis.

## 1. Introduction

The commonly held view that mitosis and meiosis are the universal forms of cell division is incomplete—some cells can also divide without the intervention of the nuclear spindle following direct nuclear fission, a process known as amitosis. The existence of amitosis has been repeatedly called into question. Many of its early accounts (e.g., [1]) have been disproved [2], its occurrence considered a rare exception [3], an aberrant or degenerative process [4], or a form of nuclear division strictly uncoupled from cell proliferation [5] and of uncertain functional significance. Since then, various forms of “true” amitosis have been documented across eukaryotes including insects [6,7], plants [8], and more tentatively, vertebrates [9,10]. Most notably, in ciliates, amitosis has evolved into the predominant means of somatic nuclear reproduction during cell proliferation [11].

In *Drosophila,* amitosis of polyploid cells in the intestinal epithelium may serve as a significant mechanism of de-differentiation associated with stem cell replenishment [7]. This mechanism may also initiate cancer through the formation of aneuploid cells [7]. In vertebrates, amitosis may occur in damaged or cancerous liver cells [9], or in deciduous tissues with subpopulations of polyploid cells such as the trophoblast [10]. Polyploidy, achieved through endomitosis or endoreplication [12,13], may promote DNA-damage insensitivity through various mechanisms in plants, insects, and bacteria, and serve as a virulence factor in pathogenic fungi [14]. In addition, mitotic de-polyploidization of polyploid cells is associated with cell rejuvenation in cancer [15], and, similar to amitosis, can readily generate populations of genetically heterogeneous cells (aneuploid cells) capable of rapid adaptive evolution (e.g., in response to xenobiotics or tissue damage [16,17]). Despite the widespread phylogenetic distribution of amitosis, its potential role in stem cell differentiation, and cancer onset and progression, this form of unbalanced nuclear division is severely understudied.

Ciliates offer a powerful system for gaining insights into the process of amitosis. Ciliated protozoans such as *Paramecium tetraurelia* (henceforth *Paramecium*) are characterized by two functionally specialized nuclei with distinct nuclear architectures [18]. The small diploid germline nucleus, the micronucleus, is transcriptionally silent during asexual division and harbors the germline genome. In contrast, the larger somatic nucleus—the macronucleus—is expressed during vegetative growth. Its expression governs cell physiology and behavior [19]. In *Paramecium*, the somatic genome is highly polyploid. This high-level ploidy is achieved during the biogenesis of the macronucleus through an endoreplication process, in which a copy of the diploid germline genome is used as a template for amplification (from 2*n* to ~860C [20]).

During the vegetative life of *Paramecium*, the diploid micronuclei divide mitotically, whereas the macronucleus divides amitotically—it elongates and eventually separates into two daughter macronuclei. Upon amitosis, allele segregation is subject to random fluctuations. To this day, it is not entirely clear how cells can avoid severe aneuploid imbalances over prolonged vegetative division [21]. This is especially true for the ciliate *Tetrahymena*, which has a much lower ploidy than *Paramecium* (~45C [22,23,24,25]). Although not necessarily sufficient to maintain constant ploidy levels across the genome, there is evidence that in *Paramecium* the total macronuclear DNA content is tightly regulated across divisions [26]. This hints at the existence of a compensatory “replicative control” mechanism that may occur at the individual chromosome level [19,21]. Such a mechanism would prevent aneuploid imbalance (deviations from the original ploidy). That noted, natural selection acting on chromosome copy number variation alone may be sufficient to avoid severe aneuploid imbalance during asexual reproduction [27,28]. Moreover, in ciliates with high ploidy levels and heavily fragmented chromosomes—where fluctuations in ploidy are anticipated to have small(er) fitness effects—the copy number of macronuclear chromosomes was found to be variable [29].

Due to the random assortment of genetic elements during amitosis of the macronucleus (henceforth *somatic assortment*), a biallelic locus eventually becomes fully homozygous for either of the alternative alleles. The rate at which this loss of heterozygosity occurs is primarily determined by the number (ploidy) and nature of the segregating units and the input ratio, i.e., the relative proportion of the two somatic alleles at the beginning of the clonal cycle [25,30,31]. Because ciliates’ macronuclei determine the cell phenotype, *somatic assortment* at heterozygous loci may give rise to *phenotypic assortment*—heterozygous clones eventually segregate into homozygous sub-clones stably expressing one of the two parental alleles [24,25,31,32]. *Phenotypic assortment* has been the primary tool for investigating *somatic assortment* and has greatly helped to understand the nature of amitosis in ciliates such as *Tetrahymena* [25]. However, a simple and direct approach that helps illuminate the process of amitosis that does not rely on phenotypic traits is currently lacking. Such an approach would conveniently allow researchers to investigate amitosis even in the absence of genetic markers that encode easily observable traits.

Recent findings concerning the process of soma development in *Paramecium* open a new perspective on how amitosis can be studied. Like other ciliates, the polyploid somatic genome of *Paramecium* is an extensively processed version of the germline genome, largely deprived of a considerable portion of DNA via a developmental process called programmed DNA elimination (PDE). In addition to removing transposons and other repetitive DNA elements, PDE removes tens of thousands of intervening, typically short (<150 bp) and AT-rich germline DNA elements termed internal eliminated sequences (IESs) [19,33,34,35]. Although IESs are, for the most part, perfectly removed from the newly developed somatic genome, some are incompletely excised—in the order of a few hundreds in standard cultivation conditions [36]. These retained elements, which we termed somatic IESs, interrupt a variable fraction (henceforth retention levels) of the total number of macronuclear DNA copies [33,36,37,38,39]. The retention levels of somatic IESs provide a measurable molecular marker to assess the random assortment of segregating alleles in *Paramecium* (and other ciliates). More explicitly, by recording the retention levels of somatic IESs across subsequent amitotic cycles (i.e., asexual generations), it should be possible to directly test the extent to which amitosis impacts the segregation of somatic alleles.

Single-cell sequencing technology (scDNA-seq) is a very convenient method to test this idea. However, the reliable detection of amitosis-associated changes in allele frequencies necessitates deep and comprehensive genome coverage as well as sensitivity and faithfulness. After individual cell isolation, scDNA-seq protocols invariably involve a step of extensive whole-genome amplification (WGA) followed by library construction and next-generation sequencing of the amplification products. Depending on the specific amplification technology and application, the WGA step can produce a satisfactory representation of the target genome [40,41]. However, WGA may also result in amplification artifacts, such as overrepresentation of large templates [42,43], reduced genome coverage [44], misrepresentation of copy number variants under certain conditions [45] (see ([46])), poor scaffold assembly [47], and allele dropout [48]. Current commercially available kits for non-PCR-based single-cell WGA minimize amplification artifacts through a highly optimized isothermal multiple displacement amplification (MDA) reaction [41,49]. Although MDA-based WGA is far more resilient to genome representation biases compared to thermocycling methods [50], it may preferentially amplify GC-rich regions [42] and lead to an under-representation of AT-rich regions (e.g., *Paramecium*’s IESs). This “selection bias” is anticipated to reach concerning levels in organisms whose genome composition lies at the low end of the GC-spectrum, such as fungi, amoebas, apicomplexans, and ciliates [51]. Potential caveats aside, scDNA-seq could be a powerful tool to trace stochastic evolution in amitotically-dividing cells.

Here, we leverage *Paramecium* to directly investigate the genetic consequences of amitosis. To this end, we first examine the suitability of single-cell sequencing to study the AT-rich genome of *Paramecium*. We then use scDNA-seq to track somatic assortment of developmentally acquired somatic mutations (retained IESs) up to 50 amitotic divisions post self-fertilization. Last, we contrast empirical estimates of *somatic assortment* with in silico predictions obtained through simulations. Collectively, the approach presented in this study forgoes the use of phenotypic markers to study the genetics of amitosis and could facilitate the identification of environmental and genetic factors affecting amitosis in a wide variety of amitotic species.

## 2. Materials and Methods

### 2.1. Paramecium Strain and Culture Conditions

Cells of *Paramecium tetraurelia* stock d12 were propagated at 25 °C in depression slides through the daily re-isolation technique (isolation culture) [30,52]. Briefly, for every passage of daily re-isolation, 200 µL of bacterized Cerophyl Medium (CM) was inoculated with a single *Paramecium* cell. The following day, a randomly selected cell was taken from the vegetative progeny of the isolate and, in turn, transferred to a new bacterized medium. Depression slides were covered with microscope slides (Diagonal GmbH & Co. KG, Münster, Germany) and kept in sealed humid chambers under controlled temperature (MIR-254-PE Cooled Incubator, PHC Europe B.V., Etten-Leur, The Netherlands).

The bacterized CM (0.25% *w/v* Wheat Grass Powder, WGP) was prepared fresh every second day by inoculating sterile CM with a 1:2000 dilution (50 µL/100 mL of CM) of *Enterobacter aerogenes* (EA) Hormaeche and Edwards (ATCC 35028) stocked in glycerol (OD = 0.5). EA-inoculated CM was incubated overnight at 30 °C under shaking (120 rpm). EA-CM was supplemented with 200 µL of stigmasterol (4 mg/mL, Merck KgaA, Darmstadt, Germany) before use.

Fresh CM (0.25% *w/v* WGP) was prepared with a 1/10 dilution of Cerophyl concentrate (2.5% *w/v* WGP). Chemical composition and pH were adjusted as in [53]. CM was autoclaved immediately after preparation and stored at RT. The Cerophyl concentrate (decoction) was prepared by boiling 25 g/L of wheat grass powder (Weizengras BIO Pulver, GSE-Vertrieb GmbH, Saarbrücken, Deutschland) in MilliQ water for 15 min. The suspension was cleared through a pre-filtration step on cellulose filters (Whatman™, Grade 595 ½, Cytiva Europe GmbH, Freiburg, Germany) followed by vacuum filtration on glass microfiber filters (Whatman™, Grade GF/F, Cytiva Europe GmbH, Freiburg, Germany). The concentrate was autoclaved and store at 4 °C.

### 2.2. Experiment Outline

In preparation for the experiment, paramecia were washed nine times in bacterized CM to remove contaminants. Three replicate populations were propagated in isolation culture under monoxenic conditions for 15 days and passed through autogamy (self-fertilization) thereafter. In order to isolate karyonidal clones—asexual progeny derived from a single event of macronuclear development—the ex-autogamous cell from each population was passaged twice through daily re-isolation. A single karyonidal founder was selected (the other two clones were discarded), expanded in a 3 mL mass culture, and used as a fully clonal parental population to set up the experiment.

Four replicate clonal isolation cultures (sub-clones) were used to conduct the single-cell DNA-seq time-course experiment. Single cells were collected in quadruplicates (one cell from each replicate population) during vegetative growth at five (D5), ten (D10), and fourteen (D14) days post autogamy. The clonal parental population was further expanded to isolate the macronuclear DNA from mass culture. The experimental design is summarized in Figure S1.

### 2.3. DNA Isolation and Sequencing

The somatic genome of the parental population was used as a reference to assess the genome representation biases of the scDNA-seq technology and was obtained as follows. Somatic nuclei were isolated from a caryonidal mass culture seven days post self-fertilization. ~10 μg of genomic DNA was purified from 500 mL mass culture in the early stationary phase (5 × 10^5^ *Paramecium* cells). The culture was cleaned up by filtration through eight layers of gauze, cells concentrated on a Nitex filter (Nylon-Netzfilter, 10 μm pore size, 47 mm, Merck KgaA, Darmstadt, Germany) and pelleted by centrifugation at 800× RCF for 3 min. Collected cells were stored in Volvic^®^ water for 1h before cell lysis to reduce bacterial load. Cells were homogenized in 4 mL of lysis buffer (0.25 M sucrose; 10 mM MgCl_2_; 10 mM Tris pH 6.8; 0.2% NP40) [33] by repeated crushing in a syringe barrel (20 mL, 60 × 25 hypodermic needle). Cell content was washed in 10mL of lysis buffer and macronuclei (MACs) isolated by centrifugation at 1000× RCF for 15 min at 4 °C. Isolated MACs were pre-lysed and gDNA extracted with the NucleoSpin^®^ Tissue Kit (MACHEREY-NAGEL GmbH & Co. KG, Düren, Germany) following the manufacturer’s instructions for DNA isolation from cultured cells.

Single cells were washed three times in Volvic^®^ water and subjected to whole-genome multiple displacement amplification (MDA) using the REPLI-g Single Cell Kit (QIAGEN GmbH, Hilden, Germany).

The parental somatic DNA from mass culture and the whole-genome amplification products from single cells (scDNA) were paired-end Illumina-sequenced (151 bp) on a NovaSeq 6000 platform at the Functional Genomic Center Zurich. A total of 12 scDNA samples and 1 bulk DNA sample were sequenced. One scDNA-seq sample failed (sc2_D14, mapping rate ~5%) and was excluded from subsequent analysis.

### 2.4. Amplification Biases of MDA-Based Whole-Genome Amplification

The degree and direction of *GC Bias* from DNA-seq data was evaluated as follows. SAM files were converted to binary, sorted, and indexed with SAMtools (version 1.4.1) [54]. Detailed *GC Bias* metrics were collected from mapped reads using the CollectGcBiasMetrics tool of the Picard suite (http://broadinstitute.github.io/picard/, accessed on 8 September 2021). GC bias estimates were calculated as the slope of the linear regression of normalized coverage on GC content between 9 and 50% GC (the two extreme GC content values of the *P. tetraurelia* genome). For convenience, *GC Bias* estimates are expressed as the change of normalized coverage every 10% change in GC content. For a sequencing experiment with a mean coverage of 100×, a *GC Bias* of 0.20 corresponds to an increase in coverage of 20 reads for every 10% increase in GC content.

The under-representation of scaffold ends (here dubbed *Terminal Bias*) was evaluated as follows. The 115 telomere-capped scaffolds (full-length macronuclear chromosomes) reported in [55] were selected for the terminal bias analysis. Coverage information was extracted from mapped reads using bedtools (https://bedtools.readthedocs.io/en/latest/index.html, accessed on 8 September 2021). The median base coverage of 492 kb-overlapping windows (1kb overlap) spanning 50 kb from either end of the 115 telomere-capped chromosomes was calculated for each sample. *Terminal Bias* estimates were calculated as the slope of the linear regression (which approximates the true parabolic relationship) of normalized windows coverage on distance from scaffold ends (up to 30 kb away from the termini where the increase in coverage plateaus). For convenience, terminal bias estimates are expressed as the change of normalized window coverage for every 10 kb change in distance from chromosome termini. A terminal bias of 0.30 corresponds to an increase in coverage of 30 reads every 10kb increase in distance from the chromosome ends for a sequencing experiment with 100× median base coverage.

A FASTQ file was artificially generated from *P. tetraurelia* stock 51 reference genome with ArtificialFastqGenerator [56] and included as a bias-free reference (aDNA). Multiple samples were processed using custom bash scripts. All data analyses were performed in R version 3.6.3 [57].

### 2.5. Data Preprocessing and Calculation of IES Retention Scores

Calling and quantification of retained internal eliminated sequences (IESs) from short reads were performed as follows. Paired-end Illumina reads (151 bp) were quality controlled with FastQC (http://www.bioinformatics.babraham.ac.uk/projects/fastqc/, accessed on 8 September 2021) and processed with BBTools version 38.90 (BBMap-Bushnell B.-https://sourceforge.net/projects/bbmap/, accessed on 8 Sept. 2021). Quality-trimming (ftm = 5, qtrim = r, trimq = 10) and adapter-trimming (ktrim = r, k = 23, mink = 11, hdist = 1, tpe tbo) were performed in a single pass with BBDuk.

Processed reads were aligned to *P. tetraurelia* reference macronuclear genome (*Paramecium tetraurelia* strain 51 MAC genome v1.0) and a pseudo-germline IES-containing genome (*Paramecium tetraurelia* strain 51 MAC + IES genome v1.0) with Bowtie 2 version 2.4.4 [58] using the local alignment function for paired-end reads in very sensitive mode (--very-sensitive-local). SAM files were manipulated with SAMtools version 1.12 [54]. IES retention scores (IRS) were estimated from sorted BAM files with the MIRET module of ParTIES [59] using the IES score method. IRS is calculated from reads supporting IES retention (IES^+^) and reads supporting IES excision (IES^−^), and equals the ratio of IES^+^ to the sum of IES^+^ and IES^−^. Reads spanning both IES boundaries are counted only once for the calculation of the IRS (https://github.com/oarnaiz/ParTIES/blob/master/user_manual.pdf, accessed on 8 September 2021).

Custom bash scripts were used for automated processing of multiple samples.

### 2.6. Quantification of the Measurement Error for IRS Estimates

The *random error* of the empirical estimates of IES retention scores (IRSs) was quantified as follows. Briefly, IRSs were estimated genome-wide on all 11 scDNA-seq samples by running ParTIES’ MIRET module with the *Boundaries* method (-score_method Boundaries). For each IES, the module calculates the retention scores for both IES boundaries (left and right). Low coverage IESs (SUPPORT_MAC + SUPPORT_LEFT + SUPPORT_RIGHT < 20 reads) and IESs with IRSs < 0.1 (IRS_left and IRS_right < 0.1) were removed from the set before downstream analyses. Significant differences between left and right retention levels were tested with a binomial test. *p* values were corrected for multiple testing using the Benjamini–Hochberg procedure. IESs with significantly different left and right retention levels (*Padj* < 0.05, 30 in total) were removed from subsequent analyses to exclude rare events of differential usage of IES boundaries [33,37]. IESs with no variability between left and right scores (304 in total) were also discarded as they represent short IESs whose boundaries are spanned by the same reads (which results in identical scores). A final set of 1196 IESs was used to estimate the distribution of *random errors* on empirical retention levels. For each IES, the *relative random error* of the retention level was taken as the coefficient of variation of the boundary scores (SD_bIRS_/bIRS, where bIRS is the mean boundary IRS score). The bioinformatics pipeline used to quantify the measurement error for IRSs is summarized in Figure S2.

### 2.7. Quantification of IES Dropout

*Total IES dropout* was calculated as the fraction of all known IES loci (*n* = 44,928) with read coverage equal to or lower than 20, as a minimum of 20 reads is desirable for robust estimation of IES retention levels (IRS) across most of the IRS spectrum. *Terminal IES dropout* was calculated as the fraction of all known IES loci located within 30 kb from either scaffold ends (*n* = 9986) and with a read coverage equal to or lower than 20. A *residual IES dropout*, likely unrelated to amplification biases, refers to the number of IESs missing from the mcDNA sample. This term is assumed to scale negatively with the number of read pairs mapped. For any given scDNA sample, the *residual IES dropout* was calculated as the *residual IES dropout* found in the mcDNA sample scaled on the sc/mc ratio of mapped read pairs:

*Residual dropout*_sc_ = *Residual dropout*_mc_/mapped read pairs (sc/mc) (1)

Last, IES dropout attributed to the positive GC bias was calculated as the dropout unexplained by either of the *terminal* or *residual dropout* terms:

*GC dropout = Total dropout − (Terminal + Residual)* (2)

### 2.8. Mathematical Modeling of Somatic Assortment

To model the probability distribution of mutant alleles (IES^+^ copies) across amitotic divisions, we leveraged previously published mathematical models of somatic assortment for ciliates [30,60]. The assumptions made to model the random segregation of alleles during amitosis and an in-depth mathematical treatment are provided in Appendix A.

### 2.9. Bioinformatic Simulation of Somatic Assortment

Through bioinformatic simulations, we estimated the probability distribution of mutated alleles (*P* (*X*)), its standard deviation (σ), and the fraction of heterozygous cells (*H)*, across successive asexual generations. We simulated the process for the daily re-isolation and mass culture regimes, with daily bottlenecks of 1 and 2^12^ (4096) cells (for a culture of 50 mL), respectively. The assumptions to model *somatic assortment* were identical to those made for the mathematical simulation with the *haploid model* (Appendix A, assumptions 1 to 5). The 860 binary subunits (two parental haplotypes) were represented with binary digits (bits). The simulation began with an input ratio of 0.5 (430 zeros and 430 ones). Cell division was simulated by drawing an equal number of subunits (860 bits) without replacement from a single set (G2 cell, 1720 bits), followed by partitioning into two sets (daughter cells). For each iteration (day) of simulated isolation culture (daily re-isolation), a single, randomly selected founder cell was used to start a series of four successive in silico cell divisions (4 div./day), which produced 2^4^ (16) cells. The process was repeated 2^10^ (1024) times to simulate replicate isolation cultures, for a total of 2^14^ (16,384) cells (*N* = 2^4^ × 2^10^ = 2^14^) across replicates. In contrast, for each iteration (day) of simulated mass culture, 2^10^ (1024), randomly selected founder cells (*digital inocolum*) were used to commence a series of four successive in silico cell divisions, which produced a total of 2^14^ (16,384) cells (*N* = 2^10^ × 2^4^ = 2^14^). The simulation was protracted for 200 generations.

### 2.10. Experimental Estimates of Somatic Assortment

To study somatic assortment experimentally, we sequenced the somatic genome of single cells using scDNA-seq across ~50 asexual divisions (see Section 2.2.). Cells divided, on average, ~3.5 times per day (25 °C) in all sub-clones studied. IRS values were determined experimentally at Day 5 (*gen* ~17), Day 10 (*gen* ~35), and Day 14 (*gen* ~50). To account for the amplification biases introduced by the MDA reaction, a set of somatic IESs—with IRS > 0.1 and coverage > 20 reads—shared across all 11 scDNA samples (at Day 5) was selected for further analysis (“track set”, *N* = 75).

### 2.11. Simulation of Retention Levels and Confidence Intervals

The mean retention levels measured experimentally 5 days post self-fertilization (D5, gen = 17, *n* = 4) were taken as starting retention levels (IRS0) to initiate the somatic assortment simulation. The probability distribution of the fraction of IES copies (simulated IRSs) expected at generation ~35 (D10) and ~50 (D14) was calculated individually for each IES locus in the “track set” (*N* = 75). The predicted standard deviation (σ) was calculated from the simulated probability distribution using Equation (A3) provided in Appendix A. σ was then used to construct a 95% Confidence Interval (CI95) around IRS_0_ for each of the 75 IES loci in the “track set”. Due to the high ploidy of *P. tetraurelia* (~860C), the simulated IRS probability distributions approximate the normal distribution within the ~50 asexual generations investigated in this study (for 0.1 < IRS_0_ < 0.9). Thus, the CI95 was calculated for Day 10 (Day 10−Day 5, ~17 *gen*), and Day 14 (D14−D5, ~31 *gen*) as IRS_0_ ± 2 × σ (IRS_0_, *gen*) (0 ≤ x ≤ 1), with σ being a function of IRS_0_ and the number of generations occurred (Equations (A3) and (A4) provided in Appendix A).

### 2.12. Code Availability

All in-house scripts and associated input files generated during this study can be accessed at https://github.com/biowalter/Amitosis (accessed on 8 September 2021). The software developed to simulate the random segregation of genetic elements in polyploid nuclei (senes.py) is made freely available as a Python script with a user-friendly command line interface at https://github.com/biowalter/senes (accessed on 8 September 2021) under the MIT license. Simulation parameters were chosen depending on the analysis performed. See README.md file for detailed usage information.

## 3. Results

### 3.1. Single-Cell DNA Sequencing of the Paramecium Somatic Genome

We first assessed the quality of the scDNA-seq data in terms of somatic genome representation (coverage), focusing on two common sources of coverage bias associated with multiple displacement amplification (MDA), namely genome composition (GC content) and position along the chromosomes. To this end, we compared a total of 11 single-cell samples (scDNA-seq) to a mass culture sample (mcDNA-seq) obtained from the same clone. Additionally, we used computer-generated reads (artificial DNA, aDNA-seq) produced from a *P. tetraurelia* reference genome as a bias-free reference.

**Figure 1:**
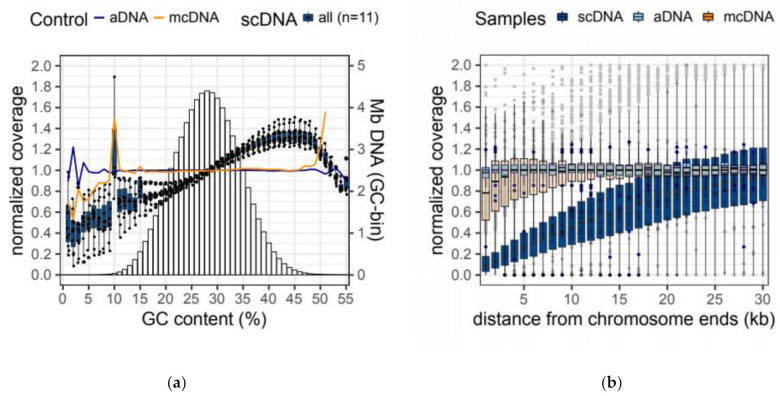
Amplification biases of MDA-based single-cell DNA seq. (**a**) *Positive GC Bias*. Change in normalized base coverage with GC content (%). Normalized coverage = n° reads/base/mean coverage. Bar chart in the background shows the amount of DNA for each GC bin (Megabases, Mb, secondary axis); (**b**) *Terminal Bias*. Change in normalized base coverage with distance from chromosome termini (kilobases, kb). Data are from 11 single-cell sequencing samples (scDNA), their parental mass culture sample (mcDNA), and one artificially generated sample (aDNA). MDA, multiple displacement amplification. scDNA, single-cell DNA sequencing. mcDNA, mass culture DNA sequencing. aDNA, artificial DNA sequencing.

mcDNA-seq and aDNA-seq show negligible GC *Bias*—i.e., a virtually homogeneous coverage across the whole range of GC-content found in *Paramecium* genome. By contrast, all scDNA-seq samples examined show a moderate positive *GC Bias*—the over-representation of GC-rich sequences (Figure 1a).

Furthermore, we find a pronounced coverage drop-off towards scaffold ends in scDNA-seq but not in mcDNA-seq data (Figure 1b). This observation is consistent with a substantial reduction of amplification efficiency during MDA at the chromosome termini. A quantitative analysis of genome representation bias for the two technologies is reported in Table 1. The genome coverage statistics for individual samples are provided in Table S1.

The bias estimates (*bGC* and *bTer*) provided in Table 1 can be used to quickly assess whether increasing scDNA-seq depth can compensate for the reduced representation of GC-poor regions and scaffold ends. For instance, a 1.5*x* increase in coverage (relative to mcDNA-seq) would fully compensate for the under-representation of ultra GC-poor regions (e.g., 20% below the mean GC content):

norm. coverage at GC_8%_ = (1 − (2 × *bGC*)) × 1.5 = 1.02, (3)

While it would not be sufficient to ameliorate the low coverage near scaffold ends (e.g., 20 kb into the reduced representation zone):

norm. coverage at 10 kb = (1 − (2 × *bTer*)) × 1.5 = 0.537. (4)

Similar observations can be made when inspecting the unnormalized coverage data (Figure S3). Collectively, we uncovered a moderate positive *GC Bias* and a severe negative *Terminal Bias* in scDNA-seq data.

### 3.2. Detection of AT-Rich Germline Sequences in the Paramecium Somatic Genome

Somatic IESs may be viewed as AT-rich insertions (somatic mutations) that occur naturally in *Paramecium* following somatic genome development [33,36,37,38,39]. To determine whether the uncovered biases of scDNA-seq (Figure 1 and Table 1) limit our ability to detect somatic IESs, we compared the somatic genomes obtained from mass culture and single cells.

**Figure 2:**
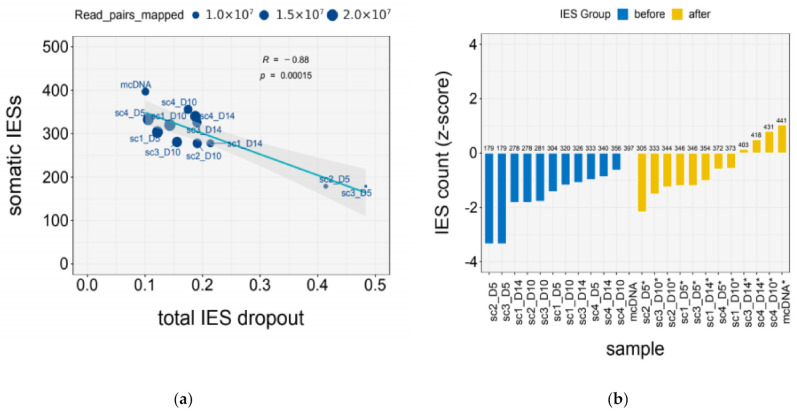
IES dropout due to uneven genome representation in scDNA samples. (**a**) Number of detected somatic IESs as a function of coverage. Number of somatic mutations detected as a function of Total IES dropout (“invisible” IES loci) and number of read pairs mapped (dot size). Somatic IESs ~ Total IES dropout, *r* = 0.882, *p* < 0.01.; (**b**) count of somatic IESs before and after correction. Somatic IES counts before and after correcting for Total IES dropout. Deviation is relative to the count obtained for bulk DNA-seq (mcDNA; *z-score* = 0). Correction, count/(1–Total IES dropout). Deviation from mcDNA count, IES count *z-score* = (IES_count–ref_value)/sd. Counts and corrected counts are indicated above bars. Sample names for corrected counts are labeled with a star sign.

Relative to the reference mcDNA, scDNA samples with a similar number of mapped reads (scDNA_1x) exhibit higher levels of IES dropout—i.e., poor or no coverage of IES-flanking macronuclear regions—due to uneven genome representation (Table 2), which can lead to the underestimation of the true number of somatic IESs in the genome (Figure 2a). However, this effect is ameliorated by an increased sequencing depth (Figure 2a)—scDNA samples with approximately double the number of mapped reads (scDNA_2x) show IES dropout levels comparable to those of the reference mcDNA (Table 2).

When we account for the level of total IES dropout, the number of somatic IESs inferred from scDNA samples more closely approximate that from the mcDNA reference (Figure 2b). Last, we tested whether IES retention levels—as measured through the IES retention score (IRS, see Methods)—are underestimated in scDNA samples compared to the mcDNA reference. Despite the elevated AT content of IESs and the detected *GC Bias* associated with single-cell DNA sequencing, we did not find evidence for preferential dropout of the mutant allele (IES^+^) (Figure S4).

Overall, our findings suggest that MDA-based scDNA-seq, when applied to an AT-rich genome such as that of *Paramecium*, can yield satisfactory genome coverage (except at chromosome termini) as long as sequencing depth is sufficiently large, ideally 1.5–2.0 fold compared to mass culture sequencing (Table S1 and Table S2).

### 3.3. IES Retention Levels across the First ~50 Amitotic Divisions Post Self-Fertilization

Next, we used the scDNA-seq data to measure the IES retention levels (IRSs) of progressively aging *Paramecium* cells. These cells had undergone ~17 (Day 5, four replicates), ~35 (Day 10, four replicates), and ~50 (Day 14, three replicates) amitotic divisions after the last self-fertilization (Figure S1). We focused on a set of 75 highly covered IES loci (reported in Table S3) for which we could accurately estimate the corresponding retention levels. Do the empirical IRS values change over time?

**Figure 3:**
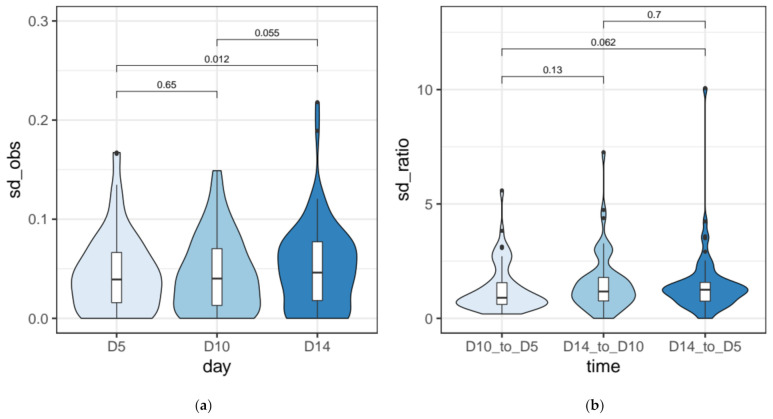
Variability of IES retention scores across time (**a**) Standard deviation of empirical retention levels (observed SD_IRS_) 5, 10, and 14 days after self-fertilization (D5, D10, D14) for a highly supported set of IESs (*N* = 75). SD_IRS_ values were computed across replicate scDNA samples (D5, *n* = 4, D10, *n* = 4, D14, *n* = 3). (**b**) Standard deviation ratios for empirical retention levels (observed SD_IRS_) for a highly supported set of IESs. SD ratios (SDR_IRS_) were computed pairwise between time points (D14 to D5, D14 to D10, D10 to D5). *N* = 60. Distributions were compared with a one-sided Wilcoxon signed-rank test. Pairwise comparisons and raw *p* values are shown above each plot.

We compared changes in the standard deviation of empirical IRS values (observed SD_IRS_), and between SD ratios (SDR_IRS_) across time points. We find a significant up-shift in the SD_IRS_ distribution over time (Figure 3a) when comparing the two points farthest apart in the time course (Wilcoxon signed-rank test, D14 vs. D5, *Padj* = 0.037, effect size r = 0.282 (small), *N* = 75). When considering the standard deviation ratios (SDR_IRS_) computed pairwise between time points, the difference is not-significant (Wilcoxon signed-rank test, D14/D5 vs. D10/D5, *Padj* = 0.187, effect size r = 0.199 (small), *N* = 60), although the density plots suggest an up-shift in the distribution over time (median D14/D5 SDR_IRS_ = 1.25) (Figure 3b). We also report the summary statistics for the observed and predicted IRS standard deviations (Table S4).

Taken together, our empirical findings support a slight (yet significant, *Padj* = 0.037) increase in variation of IES retention levels across amitotic cell divisions.

### 3.4. Simulation of Somatic Assortment

**Figure 4:**
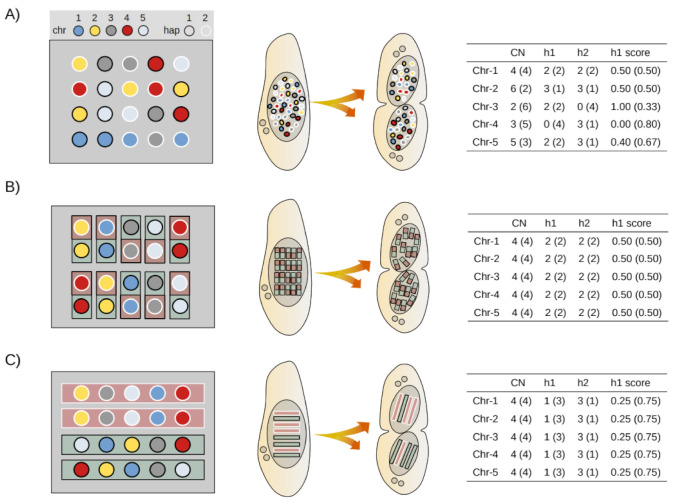
Models of macronuclear architecture in ciliates. Models for a hypothetical tetraploid cell with five somatic chromosome types (*Chr*) generated by conjugation (ex-conjugant). Left. Configuration of macronuclear sub-units in G1 (prior to DNA replication). Center. Random segregation of sub-units during amitotic division. Right. Copy number variation of individual chromosomes and their haplotypes after a single cell division. (**A**) Chromosomal model. Individual somatic chromosomes segregate freely. *N* = 2 × *Chr* × *k*, where *N* is the total number of segregating sub-units at cell division and *k* is the ploidy level.; (**B**) Diploid model. Homologous chromosomes are bundled up into diploid sub-units. *N* = *Chr* × *k*; (**C**) Haploid model. Full sets of chromosomes are bundled into single haploid sub-units. Each sub-unit contains a full complement of chromosomal variants from either one of the parental haplotypes (but not both). *N* = *2* × *k*. *CN*, Copy Number. *h1*, *CN* of haplotype 1. *h2*, *CN* of haplotype 2. *h1 score*, nuclear prevalence of haplotype 1. *h1* = *h1*/(*h1* + *h2*). Before cell division, *CN* = *k* and *h1* = *h2* = 0.50 for each chromosome type. Each daughter cell receives exactly half of the sub-units (*N*/2) at cell division (number of sub-units in G1). All chromosomes are depicted as heterozygous for illustration purposes only.

Historically, several models of ciliate macronuclear architecture have been proposed to account for observed rates of phenotypic assortment, the relative difference in DNA content between micro- and macronuclei, the absence of visible mitosis, and the avoidance of aneuploid imbalance. Figure 4 provides an overview of three distinct macronuclear configuration models and their implications.

Briefly, the *chromosomal model* assumes that individual somatic chromosomes segregate independently from each other at cell division (Figure 4A), whereas the *diploid model*, originally proposed to explain the apparent lack of allelic assortment in *Paramecium* [60,61,62], posits that homologous chromosomes (or set of chromosomes) are bundled into diploid sub-units (Figure 4B). Finally, the whole-genome haploid sub-unit model (hereafter the haploid model) hypothesizes that full sets of chromosomes from either one of the parental haplotypes are held together into larger segregating sub-units (Figure 4C). The *haploid model* is hardly conceivable for hypotrichous ciliates, whose germline chromosomes are fragmented into thousands of gene-sized mini-chromosomes during MAC development [63], and studies in *Tetrahymena* have shown that alleles assort independently unless present on the same chromosome [64,65].

Against this background, we sought to provide evidence for assortment in *Paramecium* by studying the trajectory of alternative forms (IES^+^ and IES^−^) of the same allele after self-fertilization. Following partial IES retention, the assortment of IES^+^ and IES^−^ copies across successive amitotic divisions will eventually make the macronucleus “homozygous” for either one of the two IES forms (IRS = 1, only IES^+^ copies or IRS = 0, only IES^−^ copies). But how rapidly would this loss of “heterozygosity” occur given the high ploidy levels of *Paramecium*? We first used mathematical modeling to determine how the fraction of mutant copies (IES^+^ copies) in the somatic nuclei is expected to change across successive amitotic divisions at individual IES loci. We simulated *somatic assortment* using the *haploid* and *chromosomal models* published by John Preer Jr. in 1976 [60]. We used similar parameters, except for the number of somatic chromosomes, which was then assumed to be ~43 [60], that we now know to be much larger due to chromosome fragmentation during DNA elimination. We set this parameter to 115, as there are 115 telomere-capped chromosomes in the *Paramecium* genome annotation [55]. Our predicted values correspond with those published by Preer (Table S5). To further validate our mathematical predictions, we modeled somatic assortment for mass culture and daily re-isolation through bioinformatic simulations (See Section 2.9). Mathematical and bioinformatic modeling have identical outcomes (Figure S5). The allele frequency variance for a small number of daily re-isolated lines follows a stochastic trend across generations. However, the average run for a large number of isolation cultures converges on the mathematical/mass culture predictions (Figure S5). We provide new equations to calculate the standard deviation of allele frequency distributions (e.g., retention levels) and the rate of somatic assortment (*dσ/dt*) as a function of the number of asexual divisions and starting retention levels (Equations (A5) and (A6), Appendix A).

**Figure 5:**
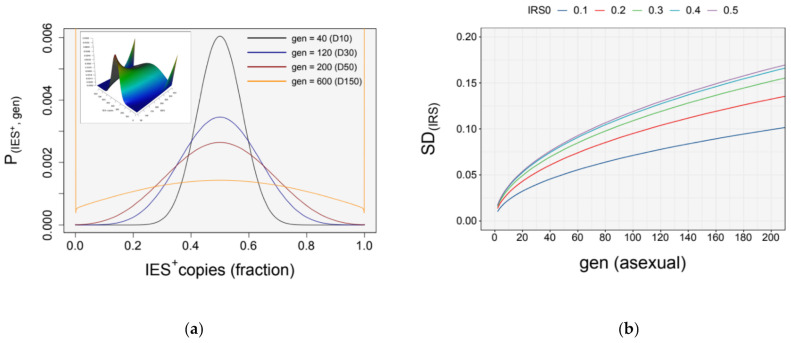

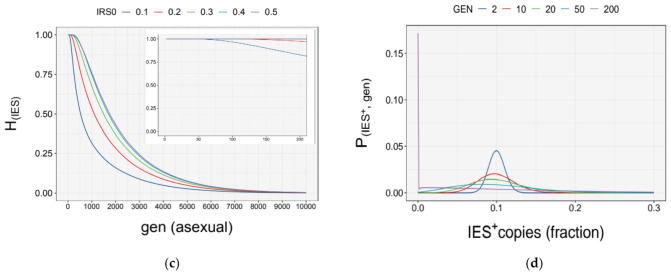
Simulations of somatic assortment for *P. tetraurelia*. (**a**) Probability distribution of IES^+^ copies, P (IES^+^; GEN). Simulated probability distribution of the number of IES^+^ copies in the somatic nuclei after successive amitotic divisions (GEN = 40, 120, 200, 600). Cultivation days (D) are indicated in brackets. The number of IES^+^ copies is expressed as a fraction of the ploidy (k = 860 (C)). Simulation is shown for IRS_0_ = 0.5. The inset shows the probability surface across generations; (**b**) Effect of assortment on standard deviation, σ (IRS_0_; GEN). Variability of the number of IES^+^ copies due to somatic assortment. The rate of somatic assortment (*dσ/dt*) is the fastest at IRS_0_ = 0.5 and decreases symmetrically around this value; (**c**) loss of heterozygosity, *H*. Probability of a locus to be in the heterozygous state across divisions. The inset shows the loss of *H* for an IES locus across a full clonal cycle of *P. tetraurelia* (average maximum lifespan of ~200 divisions [66]); (**d**) probability distribution of IES^+^ copies, P (IES^+^; GEN) across amitotic divisions (GEN = 2, 10, 20, 50, 200). Simulation is shown for IRS_0_ = 0.1. Calculations are according to the *haploid model*. IRS_0_, starting retention levels. GEN, asexual generations. In (**b**,**c**), simulated values are identical for IRS_0_ = [0.1 | 0.9; 0.2 | 0.8; 0.3 | 0.7; 0.4 | 0.6].

As expected, the simulation predicts an increase in variability of the copy number distribution of IES forms (e.g., IES^+^/IES^−^ copies) across generations (Figure 5a).

The simulated rate of *somatic assortment* peaks at an input ratio of 0.5 (starting retention level, IRS0 = 0.5), and decreases symmetrically around this value (Figure 5b). But how long would it take for cells to experience a substantial loss of heterozygosity as a consequence of the random segregation of alleles/forms at cell division? The simulation predicts that with a starting retention level (IRS_0_) of 0.5, after 200 asexual divisions—which corresponds to the average maximum clonal lifespan of *Paramecium* [66])—all cells would still be “heterozygous” for the two forms (IES^+^ and IES^−^ copies co-existing in the same nucleus) (Figure 5a,c). In fact, somatic assortment of IES^+^ and IES^−^ forms would only lead to a substantial loss of heterozygosity (e.g., *H* << 0.5) after thousands of asexual generations (Figure 5c). Furthermore, even when starting from IRS0 = 0.1 (or 0.9) the probability that an IES locus becomes fully “homozygous” after 200 divisions is smaller than 0.20 (Figure 5c, inset, Figure 5d). In summary, our simulations predict that IES retention levels remain fairly stable during asexual division, even in the presence of assortment.

Could somatic assortment give rise to *phenotypic assortment* in *Paramecium*? To address this question, we calculated the fraction of “heterozygous” (IES^+^ and IES^−^ forms of the same allele) cells that after 200 generations would undergo a “phenotypic switch” due to *somatic assortment* of IESs. Assuming an incomplete dominance scenario, wherein gene inactivation occurs when the fraction of IES+ copies exceed 0.85 of the ploidy, only ~1.4% of the cells (~6.4% under the *chromosomal model*) would express the phenotype after 200 divisions (cumulative fraction of cells with IRS > =0.85 after 200 generations). These computations refer to single loci. The probability of observing *phenotypic assortment* increases when considering multiple “heterozygous” loci simultaneously (roughly estimated by 1 − (1 − p)^n^, n = number of loci, [60]).

Taken together, our simulations suggest that the high ploidy level in *Paramecium* is associated with a modest rate of somatic assortment, which in turns prevents phenotypic assortment to take place within a short clonal cycle.

### 3.5. Somatic Assortment in Paramecium: Comparing Theoretical and Empirical Observations

**Figure 6:**
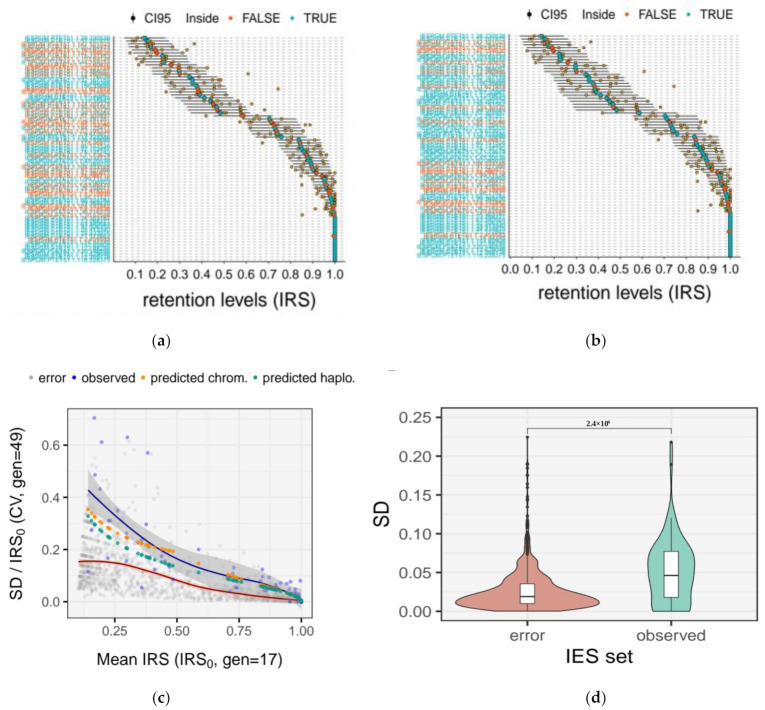
Comparison of observed and theoretical variation of IES retention levels ~50 amitotic divisions post self-fertilization. (**a**) *Haploid model*. The empirical distribution of IES retention levels is compared to the theoretical distribution predicted by the *haploid model* (random assortment of haploid whole-genome subunits); (**b**) *Chromosomal model*. The empirical distribution of IES retention levels is compared to the theoretical distribution predicted by the *chromosomal model* (random assortment of chromosomes). Retention levels (orange-filled squares) were measured experimentally with scDNA sequencing 14 days post autogamy (D14, *n* = 3) for a selected set of highly covered (>20 reads) somatic loci (“track set”, *N* = 75). Horizontal black bars represent the theoretical 95% Confidence Interval (CI) constructed on the mean retention levels (IRS_0_, large red- or green-filled circles) measured 5 days post autogamy (D5, *n* = 4), ~31 asexual generations prior. Filled circles (IRS_0_) are colored in green when the experimentally determined retention level lies inside the 95% CI for all three replicates and red otherwise. IRS, IES retention score; (**c**) observed relative variation of IRSs 14 days post self-fertilization. For each IES, the coefficient of variation of the IRSs measured on day 14 (SD_IRS_/IRS_0_, gen = 49) is plotted against the mean IRSs measured on day 5 (IRS_0_, gen = 17). *N* = 75. Predicted IRSs are shown in yellow and green for the *chromosomal* and the *haploid model* simulation, respectively. The distribution of IRS errors is shown for reference (gray circles). Local polynomial regression is shown in red and blue for the error and the empirical distribution, respectively; (**d**) comparison of measurement errors with observed IRSs. The absolute random error (SD_bIRS_) on IRS estimates (*N* = 1196) is compared to the observed variability of IRSs (SD_IRS_) measured 14 days post self-fertilization (gen = 49, *N* = 75). Distributions were compared with a Wilcoxon rank-sum test. The *p*-value is shown above the plot.

Finally, we compared the experimental dispersion of IES retention levels measured empirically ten (D10) and fourteen (D14) days post self-fertilization with that predicted in silico. For the simulations, we adopted two models of macronuclear architecture, the *haploid* and the *chromosomal model*, which predict slightly different rates of somatic assortment (See Section 2.8. for details and Figure 4). We find that on Day 14, the experimental IRS values for the aforementioned “track set” of 75 highly covered IES loci are slightly more variable than expected, regardless of the model adopted in the simulations (Figure 6a,b).

Namely, ~87% (195/225) and ~90% (202/225) of the empirical IRS values fall within the 95% confidence interval (CI95) predicted by the *haploid* and *chromosomal model*, respectively. We find a similar discrepancy between observed and predicted values on Day 10 (Figure S6).

In addition, we find that the variability of the experimental IES retention levels (expressed as coefficient of variation) changes with starting retention levels (IRS_0_) (Figure 6c), consistent with our simulations (Figure 5b) and with the random assortment of IES forms. We verified that the relative measurement error for IRSs alone is not sufficient to account for the observed variation in the empirical IRS estimates (Figure 6c,d and Figure S7). More specifically, the observed IRS variation measured 14 days post self-fertilization (gen = 49, *N* = 75) is significantly greater than that from the random error (Wilcoxon rank-sum test, *p* = 2.4 × 10^6^, effect size r = 0.132 (small), Figure 6d).

Collectively, in agreement with theoretical expectations, our empirical findings are consistent with a mild increase in variation of IES retention levels across successive amitotic divisions of the macronucleus.

## 4. Discussion

Aberrations in the chromosome number are generally studied in the context of genetic disorders. While catastrophic in diploids, aneuploidy is generally tolerated in polyploid cells. In fact, aneuploid imbalances may be a source of genetic heterogeneity and play an important role in cancer progression [15], stress-induced adaptation [16,17], resistance to DNA damage [14], and stem cell differentiation [7].

In ciliated protists, the polyploid macronucleus divides amitotically—i.e., through direct nuclear fission and without the intervention of the nuclear spindle. Thus, ciliates are a particularly amenable system for the development of accurate and scalable genomic approaches to study unequal chromosome segregation and its genetic consequences. Following DNA replication, chromatin subunits segregate randomly during amitosis [24,30,32,60]. It follows, that the nuclear frequency of an allele in heterozygous clones will change over successive asexual divisions due to stochastic segregation, which may eventually result in the production of homozygous lines with different phenotypes, a phenomenon known as *phenotypic assortment*. Traditionally, insights into the genetics of amitosis have been obtained by assessing the rate of *phenotypic assortment* [25,31]. Though powerful, this experimental approach relies on the availability of phenotypic markers. Here, we leverage *Paramecium tetraurelia*, a ciliate that houses ~860 genome copies in its somatic nucleus [20], to assess the suitability of single-cell whole-genome sequencing for directly investigating the genetics of amitosis. Re-examining the impact that amitosis may have on the somatic variability of *Paramecium* is relevant and particularly timely, as it is now clear that potentially heritable somatic variability in *Paramecium* can spark from a fully homozygous state as a consequence of incomplete excision of germline DNA sequences [36,39]—any progressive enrichment of somatic variants (e.g., retained IESs) during vegetative growth could further be transmitted to the sexual progeny. Understanding these dynamics could help reveal how the somatic rearrangement program of *Paramecium* may continuously evolve and adapt, in concert with germline mutations. Beyond *Paramecium*, the evolutionary implications of unbalanced nuclear division in polyploid asexuals are far-reaching—i.e., amitosis may sidestep or ameliorate some of the disadvantages associated with asexual reproduction (e.g., accumulation of deleterious mutations), providing benefits similar to sex [67,68].

We first explored the extent to which multiple displacement amplification (MDA) coupled with DNA sequencing (which we refer to as scDNA-seq) can be used to faithfully represent the genome of single *Paramecium* cells. We examined genome sequencing data from bulk DNA-seq of mass culture (mcDNA-seq) and single *Paramecium* cells obtained from the same clone. We find that the scDNA-seq of the *Paramecium* AT-rich genome is affected by moderate positive GC Bias (Figure 1a, Table 1 and Figure S3). We also uncover a severe sequencing coverage drop-off near chromosome ends (Figure 1b, Table 1 and Figure S3), which we refer to as negative terminal bias. The latter is consistent with the inefficient amplification of template termini in MDA reactions catalyzed by the φ29 DNA polymerase [42,69]. This terminal bias could be leveraged to determine the reproducible fragmentation patterns of ciliate chromosomes, and/or complement information from telomeric repeats to confirm full-length chromosomes in genome assemblies. In this context, the preferential amplification of large DNA templates in MDA reactions was successfully exploited to preferentially amplify the germline genome of ciliates with highly fragmented somatic DNA [43]. Finally, we show that these genome representation biases may result in the underestimation of the number of somatic IESs from single-cell samples (due to the dropout of IES loci). However, this effect can be ameliorated by increasing sequencing depth (Figure 2, Table 2 and Table S2).

With these caveats in mind, we next assessed the feasibility of tracking *somatic assortment* of somatic mutations (IES^+^) across ~50 asexual generations in single *Paramecium* cells. We tested the degree to which IES retention levels of a “track set” of 75 highly covered loci diverged after 19 and 34 amitotic divisions due to somatic assortment (for a total of ~50 amitotic divisions post self-fertilization). Our experimental estimates are consistent with a progressive, albeit slow, drift in the fraction of IES^+^ copies in the nuclei (Figure 3 and Figure 6), which is further substantiated by the outcome of mathematical and bioinformatic simulations of somatic assortment (Figure 5 and Table S5). While there is unequivocal evidence of *phenotypic assortment* in *Tetrahymena* [24,25,31,32], the existence of this phenomenon in *Paramecium* is doubtful. Using macronuclear regeneration in heterozygous clones of *P. aurelia,* Sonneborn was unable to produce phenotypic assortment [62]. Moreover, Nyberg, using a copper resistance gene as quantitative trait in *P. tetraurelia,* failed to produce evidence for an assortment of copper tolerance throughout ~250 divisions [61]. However, Preer and Nyberg cautioned that higher ploidy levels (>860C) would still be compatible with random segregation of individual somatic chromosomes [60,61]. The modest levels of assortment we measured through scDNA-seq and the results of our simulations are consistent with previous indications that somatic assortment in *P. tetraurelia* proceeds rather slowly [60]. As a consequence, *phenotypic assortment* is unlikely to be observed within a single clonal cycle [60,61]—which typically does not exceed 200 cell divisions [66]—unless cells exhibit high levels of heterozygosity, which are not characteristic of this self-fertilizing species [70] with low nucleotide diversity [71,72]. Thus, our study confirms that the high ploidy of *Paramecium* prevents *phenotypic assortment* from taking place. Furthermore, our data show that IES retention levels are largely sculpted during macronuclear development, and remain almost unchanged throughout the first 50 divisions of the clonal cycle.

Although our empirical observations are consistent with modest levels of *somatic assortment* in *Paramecium* (Figure 3 and Figure 6), part of the observed variability of the empirical IES retention levels could have resulted from sources other than *somatic assortment*, including measurement errors (Figure 6 and Figure S7), and/or the progressive fragmentation of somatic chromosomes during clonal senescence [73].

Overall, our work provides a phenotype-agnostic approach to investigate the biomedical and evolutionary significance of unequal chromosome segregation in polyploid cells. Moreover, the genome representation biases associated with multiple displacement amplification described herein may inform future studies exploring the evolution and structure of fragmented AT-rich genomes through single-cell DNA sequencing.

## Figures and Tables

**Table 1:** Quantitative analysis of genome representation. *GC Bias*. Linear regression of normalized coverage on GC content. *GC Bias* estimates are expressed as change of normalized coverage every 10% change in GC content. Normalized coverage is shown for DNA with GC content one standard deviation (sd) above (~22%) and below (~34%) the mean (28% GC). Perc., percentile. *b*, regression coefficient. *Terminal Bias*. Linear regression of normalized coverage on distance from chromosome ends (every 10kb). True relationship is parabolic. Normalized coverage is estimated for regions that are 1 and 30 kb away from either chromosome ends. *Terminal Bias* was calculated on the 115 telomere-capped chromosomes of *P. tetraurelia*. aDNA-seq, artificially generated DNA sequencing. mcDNA-seq, mass culture DNA sequencing. scDNA-seq, single-cell DNA sequencing. Mean ± sd of the mean is shown for 11 scDNA-seq samples.

Sample	GC Bias			Terminal Bias		
		Coverage			Coverage	
	*b_GC_*	16th perc. (22% GC)	84th perc. (34% GC)	*b_Ter_*	1 kb away	30 kb away
**aDNA**	0.001	0.999	1.000	0.006	0.981	1.007
**mcDNA**	−0.009	0.985	1.010	0.059	0.830	1.000
**scDNA**	0.163 ± 0.059	0.811 ± 0.043	1.160 ± 0.036	0.321 ± 0.025	0.140 ± 0.024	1.037 ± 0.029

**Table 2:** Quantitative analysis of IES dropout. *Total dropout*. Fraction of all known IES loci (*n* = 44,928) with read coverage equal to or lower than 20. *Terminal dropout*. Fraction of all known IES loci located within 30 kb from either scaffold ends (*n* = 9986) with a read coverage equal to or lower than 20. GC dropout. IES dropout unexplained by either terminal or residual dropout is assumed to results from the positive GC Bias. *Residual dropout*. IES dropout unrelated to amplification biases found in the mcDNA sample. Mapped pairs, total number of read pairs mapped (in millions). mcDNA, mass culture DNA sequencing. scDNA, single-cell DNA sequencing. scDNA_1x, scDNA samples with approximately the same number of mapped reads compared to the mcDNA sample (5 × 10^6^ < n*°* mapped reads < 15 × 10^6^, *n* = 6). scDNA_2x, scDNA samples with approximately twice as many mapped reads compared to the mcDNA sample (n*°* mapped reads > 19 × 10^6^, *n* = 4). Mapped, mapped read pairs (Millions).

Sample	Mapped (M)	IES Dropout			
		Total	Terminal	GC	Residual
**mcDNA**	10.92	0.10	0.05	0.00	0.06
**scDNA_1x**	11.29 ± 3.62	0.28 ± 0.14	0.11 ± 0.02	0.11 ± 0.09	0.06 ± 0.02
**scDNA_2x**	19.67 ± 0.51	0.12 ± 0.02	0.08 ± 0.01	0.02 ± 0.01	0.03 ± 0.001

## Data Availability

All DNA reads generated in this study are openly available in European Nucleotide Archive (https://www.ebi.ac.uk/ena/browser/home, accessed on 8 September 2021) under study accession number PRJEB43365.

## Supplementary Materials

The following are available online at https://www.mdpi.com/article/10.3390/microorganisms9091979/s1, Figure S1: Experimental setup, Figure S2: Quantification of the measurement error of IES Retention Scores, Figure S3: Genome representation biases from unnormalized coverage data, Figure S4: Comparison of empirical IES retention levels between bulk DNA-seq and scDNA-seq, Figure S5: Validation of mathematical modeling through bioinformatic simulation of somatic assortment, Figure S6: Observed and theoretical variation of IES retention levels after ~35 amitotic divisions, Figure S7: Random error distribution for IRS measurements, Table S1: Genome coverage statistics for individual samples, Table S2: Genome coverage statistics (aggregates), Table S3: Selected set of 75 highly covered IES loci tracked in this study (“track set”), Table S4: Empirical and theoretical estimates of IES retention levels across asexual divisions, Table S5: Predictions of somatic assortment-generated variability in allele frequency distribution across 250 divisions.

## Appendix A

### Appendix A.1. Assumptions

For the *haploid subunit model*, we made the following assumptions:

1. The ploidy of the somatic nucleus, *k*, is assumed to be 860 (C).
2. The total number of segregating units in the nucleus, *N*, is conserved, and amounts to 2 × *k* (1720) after DNA replication.
3. Each daughter cell receives an equal number of copies, *k*, at each cell division.
4. The number of successes is a natural number ranging from 0 to *k.*
5. The process operates in a selection-free environment.

For the *chromosomal model,* we introduced the following modifications:

6. We assumed 115 somatic chromosomes (*Chr*)
7. The total number of segregating units, *N,* is conserved and amounts to 2 × *k* × *Chr* (197,800) after DNA replication.
8. Each daughter cell receives an equal number of copies, *N*/*2*, at each cell division.
9. The number of successes is a natural number ranging from 0 to *N*/2.

Both the *haploid* and *chromosomal* models were built on the assumption that the total number of segregating units is conserved and that each daughter cell receives exactly half at cell division. However, in the *chromosomal model*, the total number of copies for a given locus is free to vary—thus, the number of IES^+^ copies will slowly tend towards a third absorbing boundary (in addition to only IES^+^ or IES^−^ copies): no copies of either form (so-called nullisomic locus). As this tendency toward chromosomal loss should, on average, affect both forms to the same extent, we made the further assumption that the probability distribution of the fraction of IES^+^ copies remains symmetrical around the input ratio.

### Appendix A.2. Mathematics

The following treatment refers to the *haploid model* notation but may be extended to the *chromosomal model* when the modifications reported above are introduced. After a first amitotic generation (*G* = 1), the probability distribution *P* (*X*) of the number of IES^+^ copies (mutated form) per nucleus in the daughter cells (number of successes *x*), represented by the random variable *X*, is a function of the number of IES^+^ copies in the parental nucleus, y_0_, and the number of copies inherited (drawn) upon division, *k*. The number of IES^+^ copies in the parental nucleus (successful elements *m*) available before division (after DNA doubling) equals 2*y*_0_.

*P*(*X*) is given by the probability mass function of the hypergeometric distribution:

(A1) $$P_{\left(X=x,G=1\right)}={\left(\begin{array}{c} 2k\\ k \end{array}\right)}^{-1}\left(\begin{array}{c} 2y_{0}\\ x \end{array}\right)\left(\begin{array}{c} 2k-2y_{0}\\ k-x \end{array}\right)$$

For the following generation, *G* = t + 1, for each *x*, *P* (*X*, *t* + 1) is the summation between *y* = *x*/2 and *y* = (*k* + *x*)/2 of the product of the probability calculated in (1), denoted *P* (*X* = *y*, *t*) at *G* = *t* + 1, and the probability of receiving *x* IES^+^ copies, for the range of possible parental IES^+^ copies *y* (*x*/2; (*k* + 2)/2) from which *x* could have been drawn.

Thus, *P* (*X*, *t* + 1) becomes:

(A2) $$P_{\left(X=x,G+1\right)}={\left(\begin{array}{c} 2k\\ k \end{array}\right)}^{-1}\overset{\left(k+x\right)/2}{\underset{y=x/2}{\sum }}P_{\left(X=y,t\right)}\left(\begin{array}{c} 2y\\ x \end{array}\right)\left(\begin{array}{c} 2k-2y\\ k-x \end{array}\right),$$

For any given number of successes *x* (number of IES^+^ copies received), the number of IES^+^ copies in the parental nuclei after DNA replication, 2*y*, must have been at least *x*, as the number of IES^+^ copies inherited (*x*) is at most equal to the total number of IES^+^ copies available in the nucleus at the time of division (*x*max = 2*y*), and could have not exceeded *k* + *x*, as *x* is at least equal to the number of IES^+^ copies present in excess with respect to *k*, the number of elements inherited upon division (*x*min = 2*y − k*). Note that the theoretical equivalent of the IES retention score (IRS calculated experimentally) is given by IRS = *x*/*k*.

### Appendix A.3. Rate of Somatic Assortment

We define the rate of somatic assortment as the change in the standard deviation, *σ*, of the probability distribution of the fraction of mutated forms (IES^+^) in the nuclei across sexual generations. At generation *G* = *t*, *σ* is given by:

(A3) $$\sigma_{t}=\sqrt{E\left[\left(X_{}-\mu_{}\right)\right]}=k^{-1}\sqrt{\overset{K}{\underset{x=1}{\sum }}\left[x^{2}P_{\left(X=x,t\right)}\right]-{\left[\overset{K}{\underset{x=1}{\sum }}xP_{\left(X=x,t\right)}\right]}^{2}}$$

Within 200 divisions, *σ* (*IRS*_0_, *t*) is approximated by:

(A4) $$\sigma_{\left(IRS_{0},G=t\right)}=a\sqrt{t}\sqrt{IRS_{0}-IRS_{0}^{2}}$$

where *IRS*_0_ (the starting parental retention level) can assume values between 0.1 and 0.9 in steps of 0.1, and the parameter *a* is equal to 0.0245 (1.4201 × 0.0245 for the *chromosomal model*). Thus, for each *IRS*_0_, the (instantaneous) rate of somatic assortment is the derivative function of σ with respect to t calculated as follows:

(A5) $$f^{\prime }\left(G=t\right)=a\sqrt{IRS_{0}-IRS_{0}^{2}}\frac{1}{2\sqrt{t}}$$

### Appendix A.4. Rate of Loss of Heterozygosity

The process of somatic assortment eventually leads to the complete loss of the heterozygous state, with nuclei containing only either mutated (IES^+^) or wild-type forms (IES^−^). The rate of loss of heterozygosity due to somatic assortment is calculated as the change of the cumulative probability of the heterozygous state, *H*, across asexual generations.

*H*, at generation *t*, is given by:

(A6) $$H_{\left(t\right)}=\overset{\left(k-1\right)}{\underset{x=1}{\sum }}P_{\left(X=x,t\right)}$$

Equation (A4) is a previously unpublished mathematical equation determined through evolutionary searches performed with the A.I.-powered modeling engine Eureqa (https://www.creativemachineslab.com/eureqa.html, accessed on 8 September 2021).
