# Supplementary material for: Fifty Generations of Amitosis: Tracing Asymmetric Allele Segregation in Polyploid Cells with Single-Cell DNA Sequencing
Source: Microorganisms. 2021 Sep 17;9(9):1979. doi: 10.3390/microorganisms9091979 (PMC8467633; doi:10.3390/microorganisms9091979)
Supplement: Supplementary file 1 [file microorganisms-09-01979-s001.zip › microorganisms-1362561-supplementary.pdf]

--- Supplementary Material ---

Fifty generations of amitosis: tracing asymmetric allele segregation in polyploid cells with single-cell DNA sequencing

Valerio Vitali*, Rebecca Rothering and Francesco Catania

Institute for Evolution and Biodiversity, University of Münster, Hüfferstrasse 1, 48149 Münster, Germany

Running title: Investigating amitosis via single-cell DNA sequencing

Keywords: Amitosis, single-cell DNA sequencing, developmental variation, copy number variation, somatic mutations, somatic assortment, polyploidy

*** Corresponding Author:** Valerio Vitali, PhD, Institute for Evolution and Biodiversity, University of Münster, Hüfferstrasse 1, 48149 Münster, Germany; Email: vitaliv@uni-muenster.de

Supplementary Figures

Supplementary Figure S1

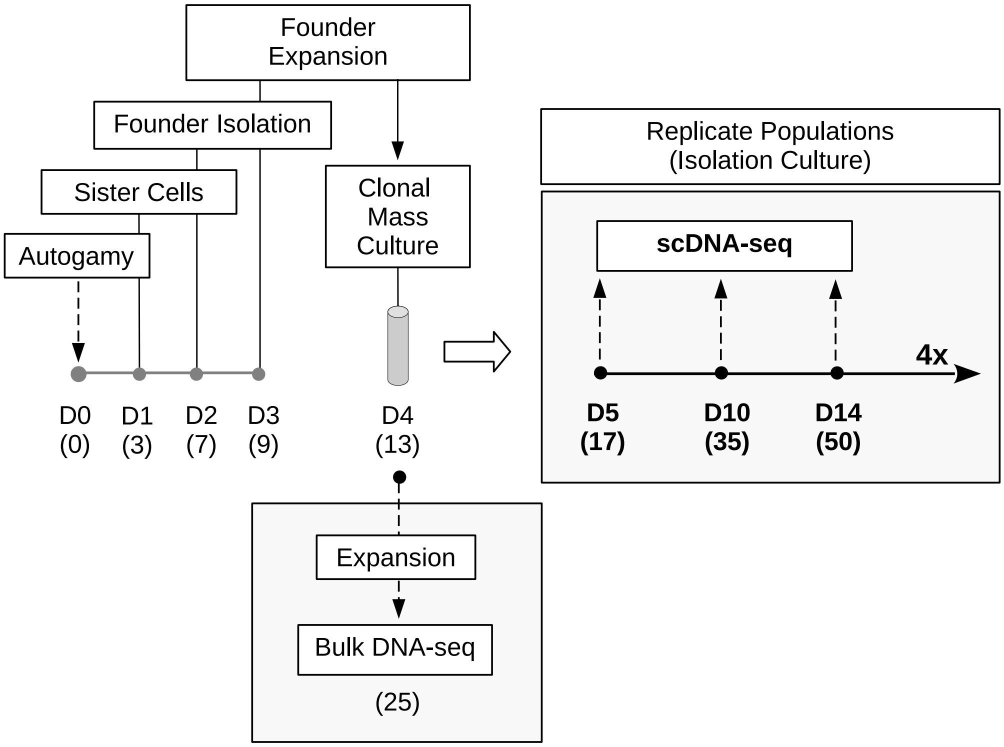

Figure S1. Experimental setup. Schematic drawing depicting the experimental setup for the time-course scDNA-seq experiment. The clonal age (divisions since last autogamy, div.) is indicated in parentheses below each day of propagation (D1 to D14). D0→D1, post-autogamous cell isolation. D1→D2, isolation of clonal founder (caryonide) at ~3 div. D2→D3→D4, expansion of clonal founder to small mass culture used to set up a series of experiments. The clonal mass culture was further expanded for bulk DNA-seq of macronuclear DNA. Clonal age is rounded to the nearest unit. For the scDNA-seq time course, the clonal age reported is the mean clonal age of the four replicate populations. The cumulative clonal age was calculated based on the divisions accrued at the time of isolation (D4→D5), and the average (across replicates and time points) of three cell count recordings taken between D5 and D14 (D5, D6 and D10; 3.73 div./24h).

Supplementary Figure S2

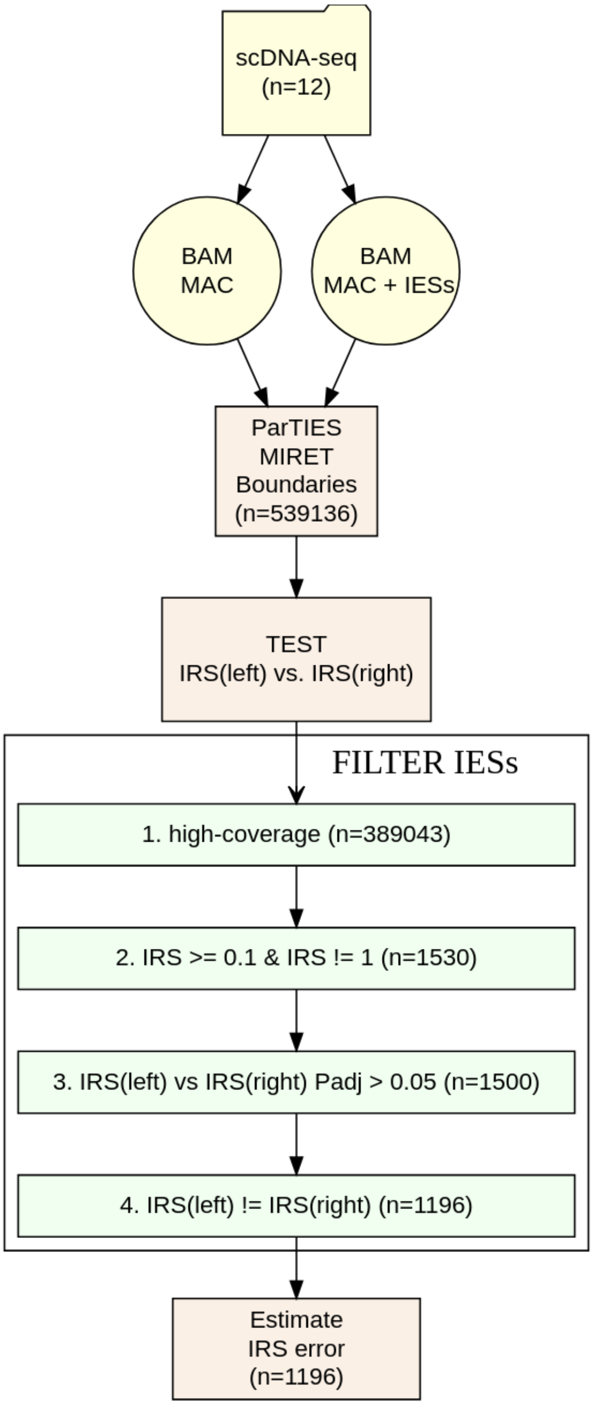

Figure S2. Quantification of the measurement error of IES Retention Scores. The diagram summarizes the bioinformatic pipeline used to estimate the random error of IES Retention Scores from scDNA-seq samples (n=12). The number of IESs passing each filtering step is indicated in parenthesis.

Supplementary Figure S3

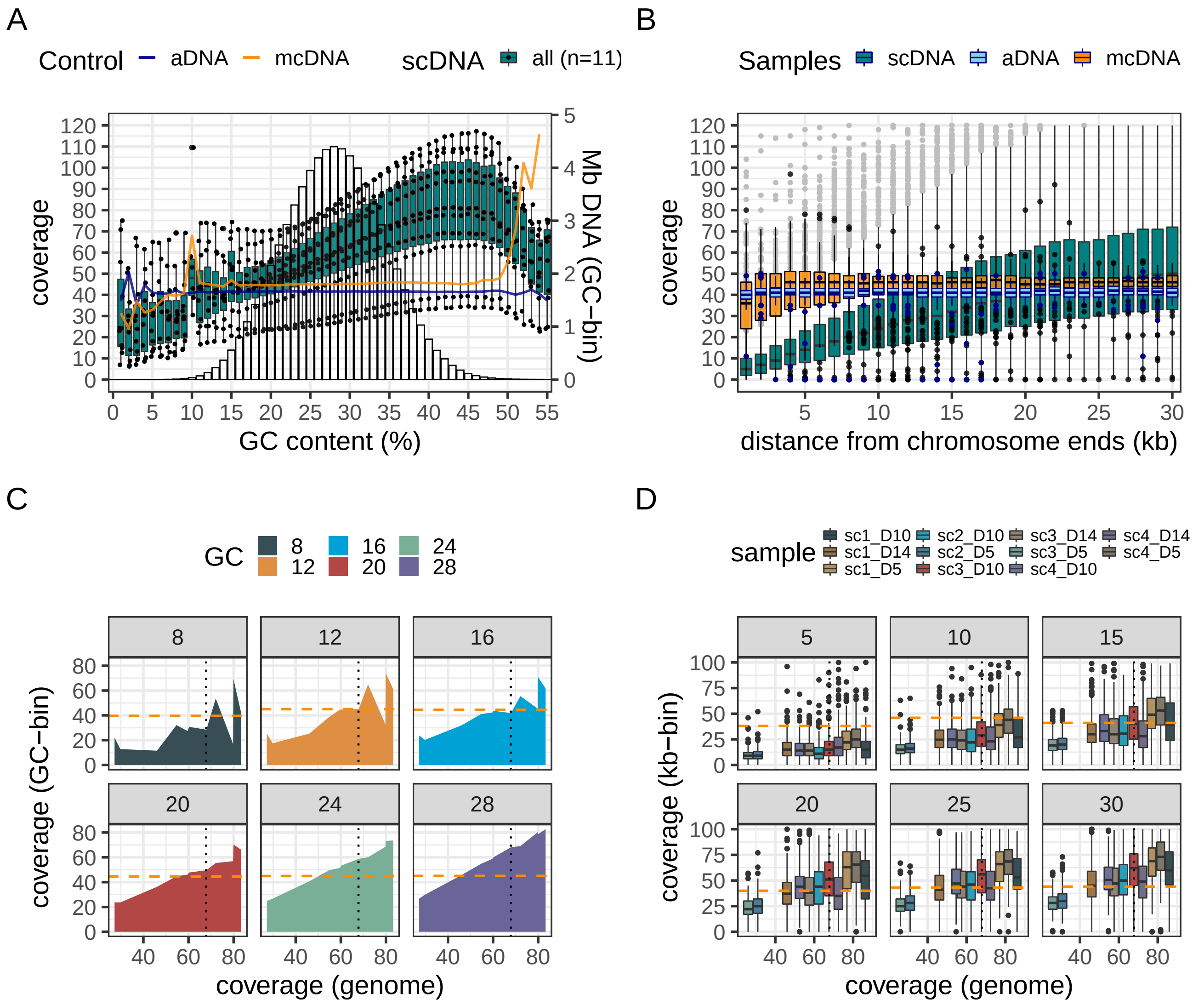

Figure S3. Genome representation biases from unnormalized coverage data. A) *Positive GC* Change in GC-bin coverage with GC content (1% bins). Bar chart in the background shows the amount of DNA for each GC bin (Megabases, Mb, secondary axis). **B)** *Terminal Bias*. Change in distance-bin coverage with distance (kilobases, kb) from chromosome termini (1kb bins). **C)** Change in GC-bin coverage with genome coverage for the lower half of the GC content spectrum (GC bin in % is shown above each facet). **D)** Change in distance-bin coverage with genome coverage (distance bin in kb is shown above each facet) up to 30kb from scaffold ends. Horizontal yellow dashed lines mark the coverage in the reference mcDNA-seq. Vertical black dotted lines mark the 1.5*x* genome coverage increase (~68) relative to mcDNA-seq (~45). scDNA, single-cell DNA sequencing (*n*=11). mcDNA, mass culture DNA sequencing (*n*=1). aDNA, artificial DNA sequencing (*n*=1). Genome coverage, average number of per-base mapped reads computed on the whole genome. GC-bin coverage, average number of per-base mapped reads computed within GC content bins. Distance-bin coverage, average number of per-base mapped reads computed within distance bins.

Supplementary Figure S4

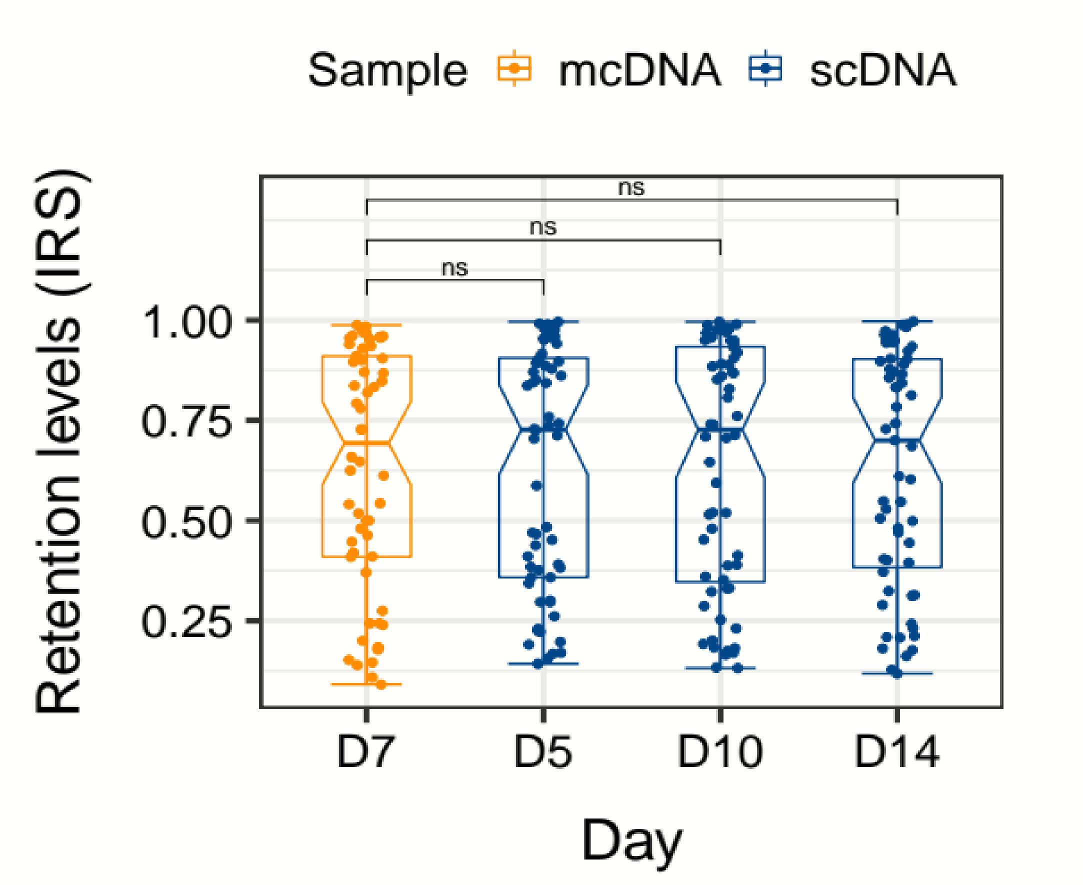

Figure S4. **Comparison of empirical IES retention levels between bulk DNA-seq from mass culture and DNA-seq from single cells.** A set of highly covered (>20 reads) somatic across scDNA samples (*n*=75, "Track Set") was selected for comparison. scDNA samples were collected in quadruplicates on each day (D5, D10, D14). IES retention levels (IRS) for scDNA samples were averaged out across replicates. IESs with IRS = 1 were excluded. IES sample size after filtering: D7 (*n*=58); D5 (*n*=60); D10 (*n*=60); D14 (n=59). IRS distributions were compared with a Wilcoxon rank sum test. mcDNA was used as reference for comparison. Significance levels for pairwise comparisons are shown above each plot (ns: *Padj* > 0.05). IRS, IES retention score / retention level. IES, Internal Eliminated Sequences. Somatic IESs, IESs from macronuclear DNA with IRS >= 0.1. mcDNA, bulk DNA-seq from mass culture. scDNA, DNA-seq from single-cell whole-genome amplification products (MDA reactions). MDA, Multiple, Displacement Amplification.

Supplementary Figure S5

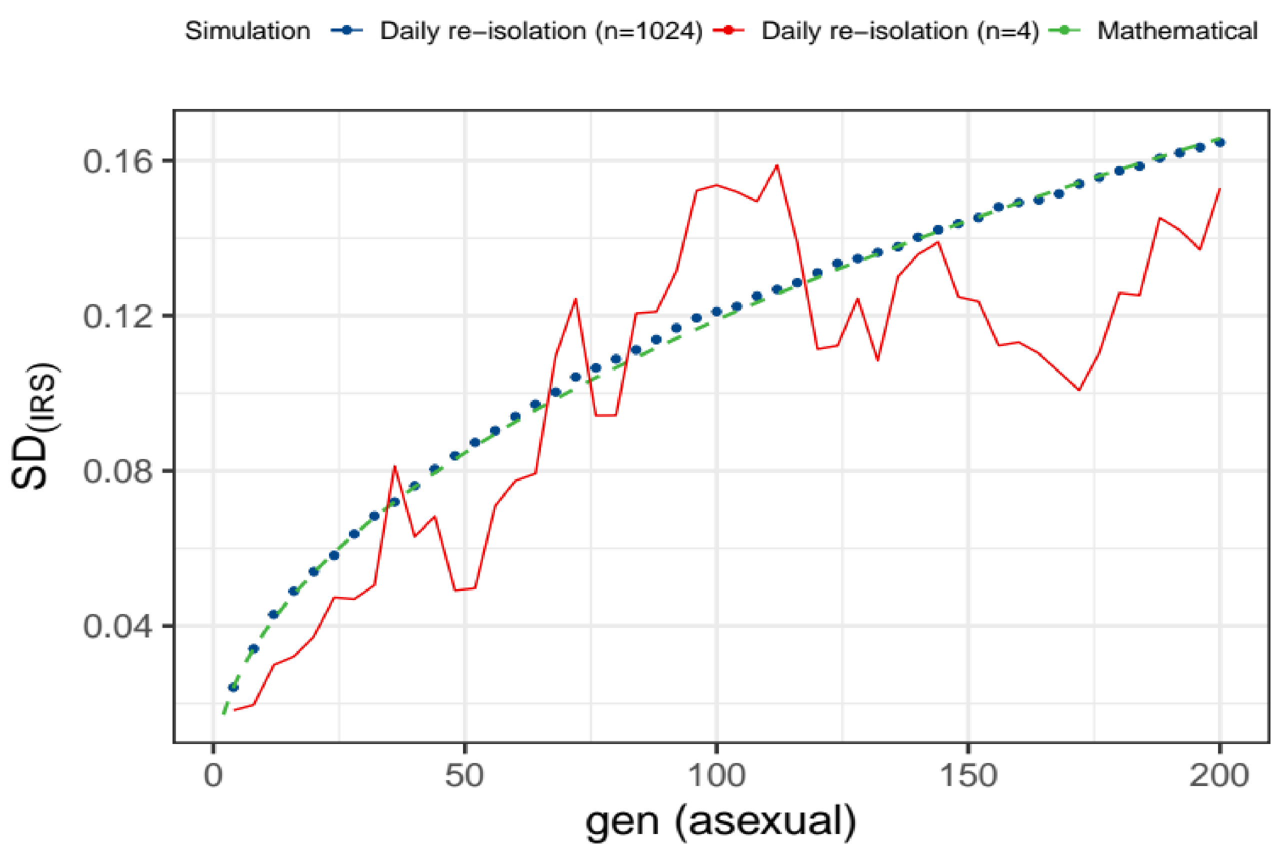

Figure S5. Validation of mathematical modeling through bioinformatic simulation of somatic assortment. Change in standard deviation (SD) of IES retention levels (IRS) across asexual generations as predicted by bioinformatic and mathematical simulations of somatic assortment. All predictions are based on the *haploid model*. See Methods for details on the simulations.

Supplementary Figure S6

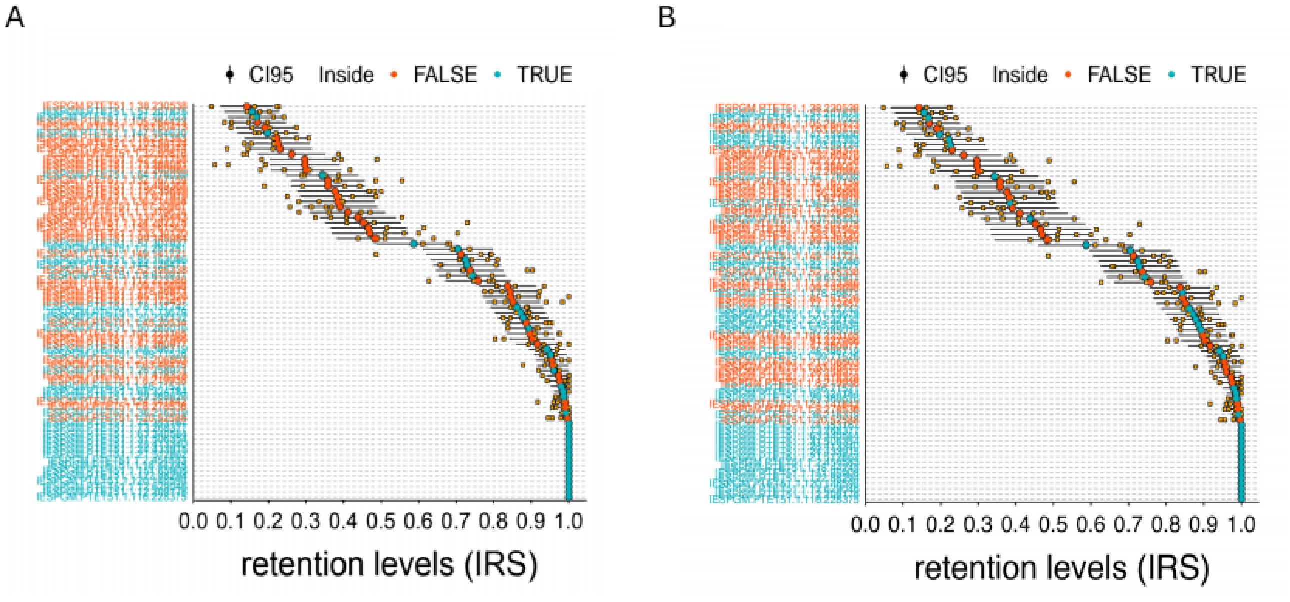

Figure S6. Observed and theoretical variation of IES retention levels after ~35 amitotic divisions. A) Haploid model. The empirical distribution of IES retention levels is compared to the theoretical distribution predicted by the haploid model (random assortment of haploid whole-genome subunits). **B) Chromosomal model.** The empirical distribution of IES retention levels is compared to the theoretical distribution predicted by the chromosomal model
(random assortment of chromosomes).

Supplementary Figure S7

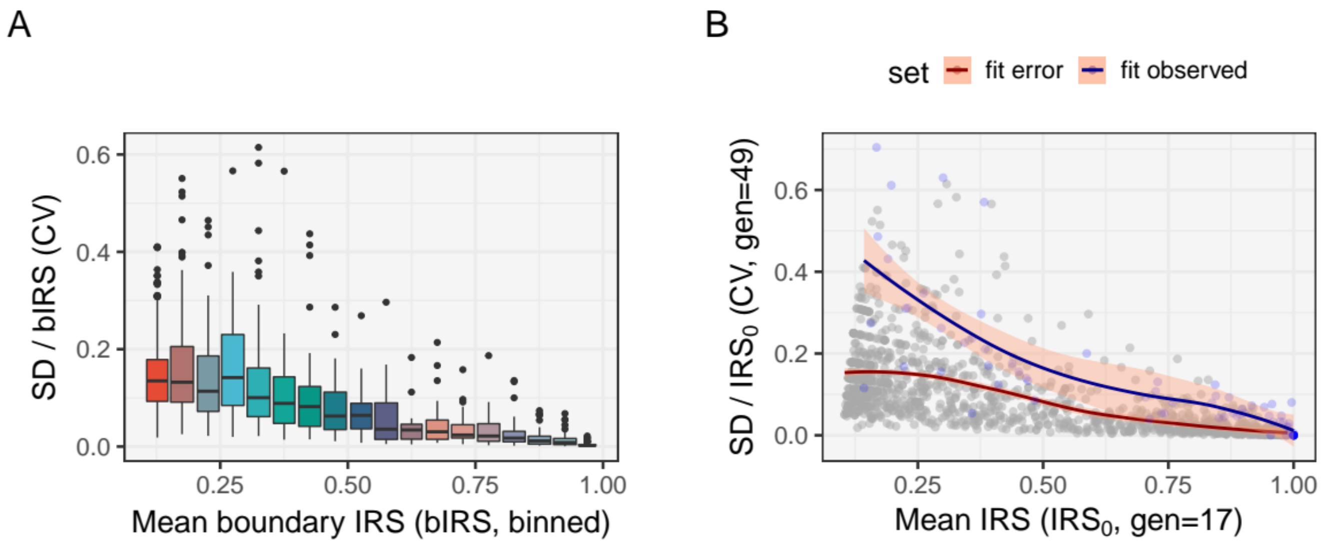

Figure S7. **Random error distribution for IRS measurements. A) Relative error of IRS measurements (binned, size = 0.05) across IRS values.** For each IES, the coefficient of variation of the boundary scores (SD_{bIRS} / bIRS) is plotted against the mean boundary score (bIRS). *N* = 1,196 (11 scDNA samples). SD_{bIRS} values were computed across left and right boundary scores (see Methods for details). Summary statistics for the relative random error distribution are: 1st Qu. = 0.0196562, Median = 0.0656980, Mean = 0.0937853, 3rd Qu. = 0.1355395. **B) Observed relative variation of IRSs 14 days post self-fertilization (blue circles).** For each IES, the coefficient of variation of the IRSs measured on day 14 (SD_{IRS} / IRS_0, gen = 49) is plotted against the mean IRSs measured on day 5 (IRS_0, gen = 17). *N* = 75 (D5, *n* = 4; D14, *n* = 3). The distribution of IRS errors (as in A) is shown for comparison (gray circles, not binned). Local polynomial regression is shown in red and blue lines for the error and the empirical distribution, respectively. Summary statistics for the absolute random error distribution are: 1st Qu. = 0.0099702, Median = 0.0189858, Mean = 0.0266522, 3rd Qu. = 0.0354437.

Supplementary Tables

Supplementary Table S1

Table S1. Genome coverage statistics for individual samples. Total pairs (M), number of read pairs (millions). **Mapped pairs (M)**, number of mapped read pairs (millions). **Mapping rate (%)**, percentage of mapped reads. **COV**, average number of per-base mapped reads. **COV 20 (%)**, proportion of bases in the genome covered by at least 20 reads (percentage). **Scaffold COV**, number of per-base mapped reads averaged across scaffolds (average scaffold coverage weighted on scaffold size). **Scaffold COV 20 (%)**, proportion of bases in a scaffold covered by at least 20 reads averaged across scaffolds (percentage). **aDNA**, artificially-generated DNA sequencing. **mcDNA**, mass culture DNA sequencing. **scDNA**, single-cell DNA sequencing.

Sample	Total pairs (M)	Mapped pairs (M)	Mapping rate (%)	COV	COV20 (%)	Scaffold COV	Scaffold COV20 (%)
**aDNA**	10.00	10.00	100.00	41.57	99.02	41.43	90.68
**mcDNA**	13.56	10.92	80.28	45.23	95.89	31.39	53.97
**sc1_D5**	20.77	19.35	92.92	79.78	92.92	39.10	44.81
**sc1_D10**	20.74	20.25	97.16	83.28	91.09	39.26	40.33
**sc1_D14**	12.13	11.21	92.11	46.06	87.66	24.11	37.04
**sc2_D5**	7.69	7.23	93.76	29.77	74.66	15.04	27.54
**sc2_D10**	15.12	14.69	96.59	60.25	88.15	30.36	37.18
**sc2_D14***	9.72	0.44	4.75	1.77	1.34	0.00	0.71
**sc3_D5**	7.87	6.62	83.90	27.23	71.48	13.76	25.51
**sc3_D10**	17.28	16.62	95.29	67.94	90.61	37.49	40.29
**sc3_D14**	15.27	14.71	94.96	59.62	88.99	34.35	38.48
**sc4_D5**	20.79	19.41	93.10	79.94	94.16	39.25	46.45
**sc4_D10**	14.04	13.27	94.11	54.67	90.49	25.47	38.34
**sc4_D14**	18.35	17.77	96.14	72.09	89.00	44.75	39.84

Supplementary Table S2

Table S2. Genome coverage statistics (aggregates). Total pairs (M), total number of read pairs (millions). **Mapped pairs (M)**, total number of mapped read pairs (millions). **Mapping rate**, fraction of reads mapped to the reference genome. **Coverage**, average number of per-base mapped reads. **Average scaffold coverage**, number of per-base mapped reads averaged across scaffolds (Coverage = average scaffold coverage weighted on scaffold size). **Coverage 20 (%)**, proportion of bases in the genome covered with at least 20 reads (expressed in percentage). **Average scaffold coverage 20 (%)**, proportion of bases in a scaffold covered with at least 20 reads averaged out across scaffolds (expressed in percentage). **aDNA**, artificially-generated DNA sequencing. **mcDNA**, mass culture DNA sequencing. **scDNA**, single-cell DNA sequencing. with a mean number of number of mapped reads comparable to the mcDNA sample (5*10^6 < *n*° of mapped reads < 15*10^6, *n*=6). **scDNA_2x**, scDNA samples with approximately twice as many mapped reads compared to the mcDNA sample (*n*° of mapped reads > 19*10^6, *n*=4).

**Sample**	**Total pairs (M)**	**Mapped pairs (M)**	**Mapping rate**	**Genome**		**Scaffolds**	
				**Coverage (reads)**	**Coverage 20 (%)**	**Average coverage (reads)**	**Average coverage 20 (%)**
**aDNA**	9.99	9.99	1.00	41.57	99.02	41.43	90.68
**mcDNA**	13.56	10.92	0.80	45.23	95.89	31.39	53.97
**scDNA 1x**	12.02 ± 3.47	11.29 ± 3.62	0.93 ± 0.05	46.27 ± 14.69	83.57 ± 8.26	23.85 ± 8.18	34.01 ± 5.87
**scDNA 2x**	20.77 ± 0.027	19.67 ± 0.51	0.94 ± 0.024	81.00 ± 1.97	92.72 ± 1.54	39.20 ± 0.089	43.86 ± 3.17

Supplementary Table S3.

Table S3. **Selected set of 75 highly covered IES loci tracked in this study ("track set"). COV**, average coverage at each IES locus (averaged across all 11 scDNA samples). **mc**, mass culture sample. **sc**, single-cell sample. **D**, day of recording.

			D5				D10				D14		
**IES_ID**	**COV**	**mc_D7**	**sc1_D5**	**sc2_D5**	**sc3_D5**	**sc4_D5**	**sc1_D10**	**sc2_D10**	**sc3_D10**	**sc4_D10**	**sc1_D14**	**sc3_D14**	**sc4_D14**
**IESPGM.PTET51.1.103.59124**	43.00	0.0909	0.2708	0.2667	0.1786	0.1864	0.1356	0.2222	0.2143	0.1538	0.1053	0.2400	0.1395
**IESPGM.PTET51.1.104.223165**	46.60	0.9583	0.9455	0.9200	0.8519	0.8696	0.9091	0.9737	0.9767	0.9388	0.8462	0.9189	0.8049
**IESPGM.PTET51.1.105.264423**	74.80	0.2745	0.1028	0.1707	0.2759	0.1308	0.0824	0.1463	0.1964	0.1017	0.1548	0.2680	0.1071
**IESPGM.PTET51.1.109.145698**	53.20	0.8704	0.8507	0.7600	0.8846	0.8523	0.9344	0.8571	0.8095	0.8077	0.8036	0.7857	0.9091
**IESPGM.PTET51.1.112.223386**	55.90	0.7800	0.7708	0.7667	0.7576	0.7391	0.7465	0.6271	0.8000	0.7872	0.8043	0.6852	0.7377
**IESPGM.PTET51.1.11.418328**	44.80	0.9296	0.9400	1.0000	1.0000	0.9524	0.9750	1.0000	0.9302	1.0000	0.9259	0.9818	0.9831
**IESPGM.PTET51.1.11.621919**	41.80	0.3704	0.4528	0.1538	0.3810	0.5417	0.2500	0.3902	0.4865	0.4237	0.4545	0.7442	0.3171
**IESPGM.PTET51.1.116.228375**	62.60	1.0000	1.0000	1.0000	1.0000	1.0000	1.0000	1.0000	1.0000	1.0000	1.0000	1.0000	1.0000
**IESPGM.PTET51.1.118.231353**	78.20	0.1837	0.2074	0.2439	0.2500	0.1875	0.2153	0.2747	0.2118	0.3077	0.2400	0.1695	0.2254
**IESPGM.PTET51.1.120.146598**	72.20	1.0000	1.0000	1.0000	1.0000	1.0000	1.0000	1.0000	1.0000	1.0000	1.0000	1.0000	1.0000
**IESPGM.PTET51.1.12.209176**	56.10	1.0000	1.0000	1.0000	1.0000	1.0000	1.0000	1.0000	1.0000	1.0000	1.0000	1.0000	1.0000
**IESPGM.PTET51.1.12.260986**	32.40	0.2000	0.1714	0.3125	0.2857	0.4318	0.3095	0.1786	0.2414	0.4167	0.6087	0.2500	0.3250
**IESPGM.PTET51.1.122.88294**	58.10	1.0000	1.0000	1.0000	1.0000	1.0000	1.0000	1.0000	1.0000	1.0000	1.0000	1.0000	1.0000
**IESPGM.PTET51.1.124.141842**	70.50	0.9111	0.9722	1.0000	1.0000	0.9881	0.9593	0.9167	0.9706	0.9846	0.9600	1.0000	0.9891
**IESPGM.PTET51.1.124.202375**	50.00	0.5405	0.4634	0.3750	0.5000	0.4684	0.4583	0.7297	0.4082	0.4808	0.6250	0.4889	0.5263

**IESPGM.PTET51.1.12.629046**	89.80	1.0000	1.0000	1.0000	1.0000	1.0000	1.0000	1.0000	1.0000	1.0000	1.0000	1.0000	1.0000
**IESPGM.PTET51.1.132.167159**	31.00	0.4091	0.5882	0.3750	0.2273	0.2414	0.4857	0.5556	0.3889	0.3793	0.4762	0.4483	0.4857
**IESPGM.PTET51.1.133.30120**	53.20	0.6122	0.6071	0.3600	0.3636	0.5484	0.7031	0.6000	0.5614	0.5116	0.6829	0.5714	0.5778
**IESPGM.PTET51.1.136.214958**	93.90	0.4800	0.3040	0.4314	0.3721	0.4307	0.3348	0.2800	0.3967	0.3971	0.3030	0.2388	0.4000
**IESPGM.PTET51.1.137.35443**	56.50	0.4634	0.4167	0.3478	0.5000	0.4881	0.3651	0.3243	0.4068	0.5556	0.5625	0.4103	0.4711
**IESPGM.PTET51.1.1.379624**	67.60	0.5000	0.4227	0.3750	0.3939	0.4512	0.4706	0.4464	0.3607	0.2807	0.4138	0.3452	0.4444
**IESPGM.PTET51.1.138.161562**	58.50	0.5179	0.5890	0.3448	0.3548	0.5769	0.4615	0.5152	0.6140	0.4694	0.4524	0.5733	0.4706
**IESPGM.PTET51.1.144.125471**	51.30	0.6471	0.6974	0.7037	0.8400	0.6739	0.8060	0.6604	0.6567	0.7308	0.6190	0.7750	0.7917
**IESPGM.PTET51.1.144.159374**	105.00	0.2432	0.2484	0.1964	0.2857	0.1895	0.1447	0.0889	0.2339	0.2111	0.1304	0.2212	0.2718
**IESPGM.PTET51.1.147.101023**	48.90	0.1458	0.1667	0.2400	0.1364	0.1270	0.2128	0.1579	0.2407	0.1562	0.1818	0.3659	0.1461
**IESPGM.PTET51.1.147.154630**	93.20	0.1818	0.2364	0.1351	0.1842	0.2324	0.2195	0.1752	0.2462	0.1628	0.3125	0.0761	0.1538
**IESPGM.PTET51.1.149.141721**	38.20	0.6250	0.7692	0.6800	0.6667	0.7333	0.7907	0.4865	0.7931	0.7667	0.6129	0.5714	0.6250
**IESPGM.PTET51.1.151.65908**	43.50	1.0000	1.0000	1.0000	1.0000	1.0000	1.0000	1.0000	1.0000	1.0000	1.0000	1.0000	1.0000
**IESPGM.PTET51.1.15.489368**	53.90	0.2432	0.3788	0.3667	0.2963	0.3902	0.4023	0.4426	0.1887	0.2909	0.2619	0.4103	0.2647
**IESPGM.PTET51.1.156.35689**	45.80	0.4468	0.4706	0.2778	0.4000	0.3571	0.4667	0.5000	0.4828	0.4688	0.4800	0.4565	0.2759
**IESPGM.PTET51.1.163.60413**	87.80	0.1389	0.1635	0.1429	0.2500	0.2051	0.2743	0.1561	0.1000	0.2025	0.3333	0.1735	0.2203
**IESPGM.PTET51.1.178.46871**	64.10	0.8475	0.8814	0.9211	0.7317	0.8396	0.8222	0.9221	0.7927	0.7761	0.8810	0.7966	0.6735
**IESPGM.PTET51.1.18.44245**	52.20	1.0000	1.0000	1.0000	1.0000	1.0000	1.0000	1.0000	1.0000	1.0000	1.0000	1.0000	1.0000
**IESPGM.PTET51.1.20.232132**	69.50	0.8367	0.9059	0.8235	0.8810	0.9036	0.9149	0.8971	0.8247	0.8444	0.8393	0.7971	0.8929
**IESPGM.PTET51.1.20.52588**	52.40	0.9688	0.9841	1.0000	1.0000	1.0000	0.9455	0.9792	0.9556	1.0000	0.8529	0.9811	1.0000
**IESPGM.PTET51.1.21.206803**	48.40	0.9800	0.9123	0.9722	0.9333	1.0000	0.9524	1.0000	0.9400	0.9811	1.0000	1.0000	1.0000

**IESPGM.PTET51.1.21.512862**	75.60	0.9877	1.0000	1.0000	1.0000	1.0000	1.0000	1.0000	1.0000	1.0000	1.0000	0.9912	1.0000
**IESPGM.PTET51.1.2.287251**	66.60	0.7925	0.6300	0.6786	0.7692	0.7391	0.7355	0.6923	0.7051	0.6901	0.6364	0.6607	0.7600
**IESPGM.PTET51.1.24.433915**	44.40	1.0000	1.0000	1.0000	1.0000	1.0000	1.0000	1.0000	1.0000	1.0000	1.0000	1.0000	1.0000
**IESPGM.PTET51.1.2.454583**	87.80	1.0000	1.0000	1.0000	1.0000	1.0000	1.0000	1.0000	1.0000	1.0000	1.0000	1.0000	1.0000
**IESPGM.PTET51.1.26.258971**	92.40	0.9841	1.0000	0.8929	0.9778	0.9681	0.9739	0.9194	0.9579	0.9750	0.9778	0.9365	0.9167
**IESPGM.PTET51.1.28.413340**	40.90	1.0000	1.0000	1.0000	1.0000	1.0000	1.0000	1.0000	1.0000	1.0000	1.0000	1.0000	1.0000
**IESPGM.PTET51.1.29.368626**	68.80	0.9615	1.0000	0.9630	1.0000	0.9836	0.9778	0.9899	0.9818	0.9643	1.0000	1.0000	0.9651
**IESPGM.PTET51.1.31.506354**	35.20	1.0000	1.0000	1.0000	1.0000	1.0000	1.0000	1.0000	1.0000	1.0000	1.0000	1.0000	1.0000
**IESPGM.PTET51.1.32.60876**	68.70	0.2391	0.2644	0.3333	0.2703	0.1772	0.3864	0.1333	0.1429	0.2609	0.1154	0.2967	0.2155
**IESPGM.PTET51.1.36.216319**	75.40	1.0000	1.0000	1.0000	0.9737	0.9929	1.0000	1.0000	1.0000	0.9833	1.0000	0.9880	0.9818
**IESPGM.PTET51.1.38.230538**	67.20	0.1778	0.1522	0.1136	0.1395	0.1647	0.1618	0.0476	0.2273	0.2222	0.1321	0.1094	0.1414
**IESPGM.PTET51.1.42.221758**	47.20	0.5429	0.2529	0.3913	0.2083	0.3333	0.4889	0.1818	0.3750	0.3939	0.4250	0.3409	0.3500
**IESPGM.PTET51.1.45.20534**	51.20	0.9048	0.8776	0.8621	0.9032	0.9032	0.9535	0.8378	0.9474	0.9388	0.8723	0.8750	0.9457
**IESPGM.PTET51.1.46.71521**	75.90	0.9556	0.9391	1.0000	1.0000	0.9794	0.9865	1.0000	0.9872	0.9839	0.9500	1.0000	0.9416
**IESPGM.PTET51.1.47.260977**	50.00	0.9400	0.8871	0.8710	0.9706	0.9362	0.9756	1.0000	0.8929	0.9333	0.8750	1.0000	0.9762
**IESPGM.PTET51.1.48.396719**	77.70	0.9600	0.8889	0.9623	0.9762	0.9386	0.9314	0.9344	0.9688	0.9167	0.8481	0.9589	0.9619
**IESPGM.PTET51.1.50.285016**	83.20	0.8961	0.8468	0.8788	0.9643	0.8819	0.8376	0.9125	0.8962	0.9178	0.8596	0.8977	0.9510
**IESPGM.PTET51.1.52.195338**	46.20	0.8333	0.7297	0.6129	0.7778	0.8243	0.7174	0.8889	0.9032	0.7188	0.7778	0.8571	0.8036
**IESPGM.PTET51.1.54.176039**	90.50	0.5000	0.3500	0.3191	0.3333	0.3707	0.3871	0.3511	0.2951	0.2879	0.3485	0.2371	0.3868
**IESPGM.PTET51.1.57.207875**	54.40	0.1087	0.1250	0.2381	0.1071	0.1538	0.2020	0.1429	0.1778	0.1765	0.0870	0.1667	0.1000
**IESPGM.PTET51.1.57.307937**	58.20	0.6585	0.5955	0.4167	0.6818	0.6543	0.6774	0.6835	0.6111	0.6111	0.6486	0.5769	0.4189

**IESPGM.PTET51.1.63.340584**	61.80	1.0000	1.0000	1.0000	1.0000	1.0000	1.0000	1.0000	1.0000	1.0000	1.0000	1.0000	1.0000
**IESPGM.PTET51.1.63.46475**	62.00	0.9355	0.9487	0.9375	1.0000	0.9412	0.9524	0.8780	0.9643	0.9362	0.8511	0.9870	0.9655
**IESPGM.PTET51.1.67.20967**	59.40	0.4103	0.3218	0.4565	0.3333	0.4512	0.3529	0.2571	0.3671	0.3137	0.3750	0.5079	0.4500
**IESPGM.PTET51.1.68.324184**	63.30	1.0000	0.9412	1.0000	1.0000	1.0000	0.9802	1.0000	0.9825	0.9865	1.0000	1.0000	1.0000
**IESPGM.PTET51.1.71.73679**	70.70	0.9697	0.8919	0.8333	0.8667	0.8919	0.8889	0.8594	0.8955	0.8947	0.9625	0.8116	0.9375
**IESPGM.PTET51.1.73.268051**	53.80	0.1522	0.3600	0.3750	0.2143	0.2375	0.1915	0.0923	0.1912	0.0571	0.3784	0.2344	0.2553
**IESPGM.PTET51.1.73.268377**	66.00	1.0000	1.0000	1.0000	1.0000	1.0000	1.0000	1.0000	1.0000	1.0000	1.0000	1.0000	1.0000
**IESPGM.PTET51.1.74.353167**	105.00	1.0000	1.0000	1.0000	1.0000	1.0000	1.0000	1.0000	1.0000	1.0000	1.0000	1.0000	1.0000
**IESPGM.PTET51.1.77.172457**	61.20	0.9000	0.8990	0.8261	0.8462	0.8333	0.8023	0.9394	0.8302	0.9831	0.9054	0.8333	0.8750
**IESPGM.PTET51.1.78.137742**	63.90	0.9091	0.8133	0.9048	0.9130	0.8154	0.8909	0.8491	0.8875	0.8444	0.9333	0.8587	0.8784
**IESPGM.PTET51.1.8.219938**	100.00	1.0000	0.9802	1.0000	0.9512	0.9708	1.0000	0.9916	0.9892	0.9421	1.0000	1.0000	0.9194
**IESPGM.PTET51.1.8.278536**	59.70	0.9535	0.9620	1.0000	1.0000	1.0000	1.0000	0.9846	0.9556	0.9661	0.9556	0.9661	0.9118
**IESPGM.PTET51.1.85.151526**	58.20	0.7273	0.8481	0.8400	0.8182	0.8732	0.8660	0.9194	0.8387	1.0000	0.9189	0.9310	0.7843
**IESPGM.PTET51.1.85.153086**	64.30	0.8194	0.7333	0.7692	0.6250	0.7711	0.8101	0.7833	0.7083	0.7414	0.8667	0.7432	0.8876
**IESPGM.PTET51.1.91.227966**	62.50	0.8679	0.9394	1.0000	0.7600	0.9091	0.9104	0.9020	0.8267	0.8039	0.8226	0.8548	0.9194
**IESPGM.PTET51.1.9.613911**	97.50	0.7273	0.7099	0.8696	0.6744	0.7153	0.7957	0.7356	0.7478	0.6790	0.7093	0.6567	0.7341
**IESPGM.PTET51.1.99.145984**	47.60	0.4194	0.4098	0.4483	0.5000	0.5758	0.4386	0.4906	0.5106	0.6400	0.5000	0.5098	0.5758
**IESPGM.PTET51.1.99.71526**	112.00	0.9565	0.9514	0.9608	0.9118	0.9808	0.9548	0.9487	0.9655	0.9278	0.9870	0.8992	0.9620

Supplementary Table S4

Table S4. Empirical and theoretical estimates of IES retention levels across asexual divisions. Empirical, observed variation in IES retention levels (IRS). Haploid, calculations based on the haploid whole-genome subunits model. Chromosomal, calculations based on the chromosomal model. Median standard deviation of IRS values is shown within brackets. Mean retention levels at Day 5 were taken as starting retention levels (IRS_0). Data are relative to 75 highly covered IES loci (> 20 mapped reads). IRS, IES Retention Scores. Day, 5, 10 and 14, ~17, ~35 and ~49 divisions after self-fertilization respectively. SD_{IRS}, observed and predicted standard deviation of retention levels.

	Time Point		
Mean SD_{IRS} (*median*)	**Day 5**	**Day 10**	**Day 14**
**Empirical**	0.0449 (*0.0392*)	0.0449 (*0.0401*)	0.0507 (*0.0461*)
**Haploid**	n/a	0.0296 (*0.0356*)	0.0394 (*0.0474*)
**Chromosomal**	n/a	0.0394 (*0.0506*)	0.0559 (*0.0673*)

Supplementary Table S5

Table S5. Predictions of somatic assortment-generated variability in allele frequency distribution across 250 divisions according to this study and Preer 1976. Predictions with the *haploid model* are for an initial number of 430 subunits and a total number of 860. Predictions with the *chromosomal model* are for an initial number of 430 subunits and a total number of 43). The standard deviation of the allele frequency is reported as fraction of the starting number of segregating subunits as in Preer 1976 (rather than fraction of the ploidy level as reported by SENES.py (this study)). The discrepancy between SENES.py and Preer's predictions for the *chromosomal model* is due to our assumption that the tendency toward chromosomal loss will affect both alleles and thus the relative fraction of IES+ copies (retention level) would remain symmetrical. To drop this assumption and reproduce Preer's predictions exactly, SENES.py should be ran with the --nullisomics flag on.

	Haploid model		*Chromosomal Model*	
**GEN**	**SD (SENES.py)[a]**	**SD (Preer 1976)[b]**	**SD (SENES.py)[c]**	**SD (Preer 1976)[d]**
50	0.16934	0.17000	0.23960	0.24000
100	0.23776	0.24000	0.33576	0.34000
150	0.28911	0.29000	0.40283	0.42000
200	0.33146	0.33000	0.45346	0.48000
250	0.36795	0.37000	0.49376	0.54000

[a] Simulation with -m haploid -k 860 -i 0.5 -g 250
[b] Data from Table 5
[c] Simulation with -m chromosomal -c 43 -k 860 -i 0.5 -g 250
[d] Data from Table 3
